# Activation of HCA2 regulates microglial responses to alleviate neurodegeneration in LPS-induced in vivo and in vitro models

**DOI:** 10.1186/s12974-023-02762-5

**Published:** 2023-03-29

**Authors:** Dewei He, Shoupeng Fu, Bojian Ye, Hefei Wang, Yuan He, Zhe Li, Jie Li, Xiyu Gao, Dianfeng Liu

**Affiliations:** 1 College of Animal Science, Jilin University, Changchun, China; 2 College of Veterinary Medicine, Jilin University, Changchun, China

**Keywords:** Parkinson's disease, HCA2, Neuroinflammation, Nicotinic acid

## Abstract

**Background:**

Previous studies have shown a close association between an altered immune system and Parkinson's disease (PD). Neuroinflammation inhibition may be an effective measure to prevent PD. Recently, numerous reports have highlighted the potential of hydroxy-carboxylic acid receptor 2 (HCA2) in inflammation-related diseases. Notably, the role of HCA2 in neurodegenerative diseases is also becoming more widely known. However, its role and exact mechanism in PD remain to be investigated. Nicotinic acid (NA) is one of the crucial ligands of HCA2, activating it. Based on such findings, this study aimed to examine the effect of HCA2 on neuroinflammation and the role of NA-activated HCA2 in PD and its underlying mechanisms.

**Methods:**

For in vivo studies, 10-week-old male C57BL/6 and HCA2^−/−^ mice were injected with LPS in the substantia nigra (SN) to construct a PD model. The motor behavior of mice was detected using open field, pole-climbing and rotor experiment. The damage to the mice's dopaminergic neurons was detected using immunohistochemical staining and western blotting methods. In vitro, inflammatory mediators (IL-6, TNF-α, iNOS and COX-2) and anti-inflammatory factors (Arg-1, Ym-1, CD206 and IL-10) were detected using RT-PCR, ELISA and immunofluorescence. Inflammatory pathways (AKT, PPARγ and NF-κB) were delineated by RT-PCR and western blotting. Neuronal damage was detected using CCK8, LDH, and flow cytometry assays.

**Results:**

HCA2^−/−^ increases mice susceptibility to dopaminergic neuronal injury, motor deficits, and inflammatory responses. Mechanistically, HCA2 activation in microglia promotes anti-inflammatory microglia and inhibits pro-inflammatory microglia by activating AKT/PPARγ and inhibiting NF-κB signaling pathways. Further, HCA2 activation in microglia attenuates microglial activation-mediated neuronal injury. Moreover, nicotinic acid (NA), a specific agonist of HCA2, alleviated dopaminergic neuronal injury and motor deficits in PD mice by activating HCA2 in microglia in vivo.

**Conclusions:**

Niacin receptor HCA2 modulates microglial phenotype to inhibit neurodegeneration in LPS-induced in vivo and in vitro models.

**Supplementary Information:**

The online version contains supplementary material available at 10.1186/s12974-023-02762-5.

## Introduction

Parkinson's disease (PD) is a neurodegenerative disease more common in the elderly, with an average onset age of around 60 years. Like neurodegenerative diseases, neurons are the main target of damage in PD, where the degeneration and death of dopamine (DA) neurons in the substantia nigra (SN) significantly decrease striatal DA content to cause the disease [1, 2]. The exact cause of PD is currently unknown. Studies have shown that PD is not caused by a single factor. Genetic factors, physiological aging, and other factors that increase susceptibility to PD, oxidative stress, calcium ion imbalance, mitochondrial failure, etc., lead to the degeneration and death of neurons, causing the disease [3–5]. Growing pieces of evidence suggest neuroinflammation plays a pivotal role in PD progression. During PD, the primary immune cells in the central nervous system (CNS), the microglia, proliferate rapidly, causing phagocytosis and eliminating harmful factors. However, continuously activated microglia release many inflammatory mediators, damage normal neurons, and aggravate the PD process [6–8]. Therefore, alleviating neuroinflammation after PD may be an essential means to alleviate the symptoms of the disease.

Hydroxy-carboxylic acid receptor 2 (HCA2), also known as GPR109A, is a Gi protein-coupled receptor ubiquitously present in brown and white adipose tissue. It was initially known mainly for its lipolytic and hypolipidemic functions. Subsequently, it was found to be abundantly expressed in immune cells such as macrophages, monocytes, and neutrophils resulting in widely knowing of its role in immune system-related diseases [9, 10]. HCA2 has three significant ligands, nicotinic acid (NA), sodium butyrate (SB) and β-hydroxybutyric acid (BHBA), which can activate HCA2 in varying degrees to play a therapeutic role in diseases. Chen et al. showed that sodium butyrate inhibits 2,4,6-trinitrobenzenesulfonic acid (TNBS)-induced colitis and activates GPR109A to maintain intestinal barrier integrity [11]. Gong et al. found that GPR109A maintains gut integrity and prevents enterotoxigenic Escherichia coli (ETEC) mucosal infection by promoting IgA secretion [12]. Guo et al. found that butyrate activates GPR109A to alleviate oxidative stress in dairy cow mammary glands [13]. In addition, our research group, Fu et al., found that BHBA inhibits neuroinflammation by activating GPR109A [14], suggesting its potential essential role in CNS diseases. However, its specific function and underlying mechanisms remain to be explored.

Nicotinic acid (NA) belongs to the B group of vitamins, also known as vitamin B3. It mainly exists in animal offal, milk, eggs, bran, and fresh vegetables. Presently, NA is primarily used as a feed additive that improves the utilization rate of feed protein. NA is a well-known pharmaceutical intermediate used as a raw material to synthesize various medicines [15, 16]. As a ligand of HCA2, NA has been implicated in numerous diseases where it activates HCA2. Guo et al. found that NA alleviates dairy cow mastitis by activating GPR109A [17]. Miguel et al. found that NA regulates microglial responses and limits disease progression by activating HCA2 in a mouse model of Alzheimer's disease [18]. Similarly, Chandramohan et al. found that NA regulates macrophage polarization by activating HCA2 in microglia [19]. However, there are relatively few studies highlighting the effect and mechanism of NA on PD.

These findings collectively show the interaction of NA and HCA2 appears in numerous peripheral and CNS diseases. However, the specific mechanism of HCA2 regulation in PD remains to be further determined. We hypothesize that HCA2 promotes a beneficial microglial phenotype in PD that can be stimulated pharmacologically by NA.

## Materials and methods

### Reagent

Nicotinic acid (NA, > 98%), dimethylsulfoxide (DMSO), lipopolysaccharide (LPS) and Lipofectamine 2000 were obtained from Sigma-Aldrich, St. (MO, USA). MK2206 (an Akt inhibitor) was purchased from Apex Bio Technology. (Houston, USA). Fetal bovine serum (FBS) was obtained from Hyclone (Logan, Utah, US). Penicillin–streptomycin (PS) solutions, Dulbecco’s and modified Eagle’s medium (DMEM) and Opti MEM were obtained from Invitrogen (Carlsbad, CA, USA). Trypsin was obtained from CHI Scientific (Maynard, MA, US).

### Animal and model

**Fig. 1:**
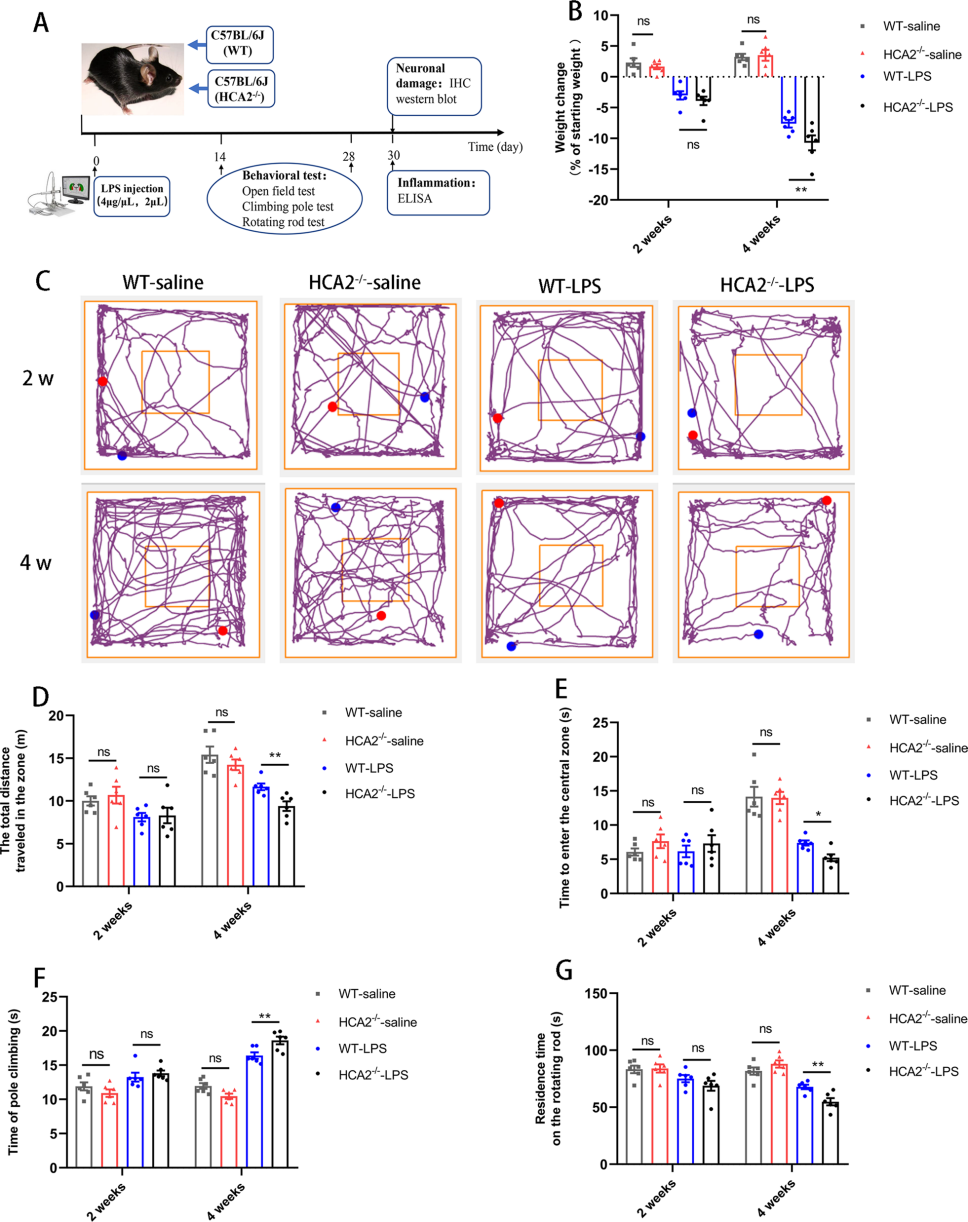
Effect of HCA2 on body weight loss and exercise capacity in mice. **A** Experimental flowchart. **B** Changes in the body weight of mice before and after the experiment. **C** Movement trajectories of mice in the open field. **D** Total distance traveled by mice in the open field. **E** Residence time of mice in the center box. **F** The time mice took for climbing the pole. **G** The time that the mice did not fall on the rotarod fatigue apparatus. Results are presented as mean ± SD (*n* = 6). *p < 0.05, **p < 0.01 means significant difference and *ns* means no significant difference

In this experiment, we used 10-week-old male wild type (WT) C57BL/6 and HCA2^−/−^ mice. The mice were obtained from Liaoning Chang sheng (Liaoning, China) and Sai ye Biotechnology Co (Guangzhou, China). All animal experiments followed the specifications of the Animal Care and Use Committee of Jilin University (Permit number: SY202107003). We set up two batches of mice for experiments. In the first batch, WT and HCA2^−/−^ mice were divided into four groups (6 mice/group): WT-LPS group (4 μg/μL, 2 μL), WT-saline group, HCA2^−/−^ saline group, and HCA2^−/−^ -LPS group. According to our previous experiments [20], we successfully established a nigral inflammation model which recapitulates some symptoms and pathology of PD with LPS injection (stereotactic coordinates: dorsoventral (DV) = -2.5 mm, mediolateral (ML) = -0.8 mm and anterior–posterior (AP) =  + 0.5 mm). After anesthetizing the mice, we injected LPS or an equal volume of saline into the right SN of the mice. On days 14 and 28 after the LPS injection, mice were subjected to behavioral testing. The experimental timing is shown in Fig. 1A. We then commissioned Saiye Biotechnology Co (Guangzhou, China) to construct conditional knockout mice with HCA2^−/−^ on microglia (HCA2^Floxp^Cxc3r1^cre^) for further studying the role of HCA2 in PD. In the second batch, WT and HCA2^Floxp^Cxc3r1^cre^ mice were also divided into four groups (6 mice/group): WT-LPS group, WT-LPS + NA group, HCA2^Floxp^Cxc3r1^cre^-LPS group, and HCA2^Floxp^Cxc3r1^cre^ -LPS + NA group. Molding was performed with the same method, followed by daily feeding of nicotinic acid (NA) (drinking water, 3 mg/mL, 6 mL/day) for 28 days. On day 28 after the LPS injection, mice were subjected to behavioral testing. The experimental timing is shown in Fig. 9A.

### Behavioral test

In the first batch of experiments, mice were subjected to behavioral testing in the second and fourth weeks after the LPS injection. In the second batch, mice were subjected to behavioral testing in the fourth weeks after the LPS injection. Behavioral tests include open-field test, rotor test and climbing rod tests.

### Open field experiment

Mice were placed in an open-field environment (50 cm × 50 cm × 30 cm) to move freely. A computer system (Stoelting Co, US) recorded the distance the mice moved in the open field and the time it took to enter the central area in 5 min. The test site was dark and quiet. It was cleaned before the start of the experiment. All mice were placed in a box before the investigation to allow them to acclimatize to the environment.

### Pole climbing experiment

After the open-field experiment, we conducted a pole-climbing investigation, where the mice were trained for 3 days before the experiment to acclimatize them. The size of the pole used was 50 cm. Our experiment recorded the time taken for the mice to climb continuously from the top to the bottom of the pole. Each mouse was made to repeat the process three times, and the mean was then calculated.

### Rotor experiment

In this experiment, the mice were trained to adapt to the investigation before the experiment. The rotating rod was 60 cm long and 30 cm in diameter. We placed the mice on the rotating rod fatigue apparatus (ZS-RDM, Zhongshi Technology, Beijing), adjusted the speed of the rotating rod for 40 rpm and started the apparatus to record the latency to fall of each mouse. It was repeated three times, and the mean value was calculated.

### Immunohistochemical staining

After euthanizing the mice, their midbrains were removed and fixed using 4% paraformaldehyde. Then the midbrain was placed in 70%, 80%, 90%, 95%, and 100% alcohol for one hour each for dehydration. Then it was dipped in xylene for 30 min and immersed in wax for 60 min. The midbrain tissue was then sectioned (6 μm per slice) and dried. Dopaminergic neurons in SN were measured with rabbit anti-TH antibody (1:500, while microglia were detected with rabbit anti-IBA-1 antibody (1:500; Abcam, Cambridge, UK). The IHC staining was performed according to the instructions of the immunohistochemistry kit (Biological Technology, Wuhan, China). Finally, total TH and IBA-1-positive cells were counted by five researchers in a blinded fashion (who were unaware of the experimental design), and the average of these scores was reported. At least 3 slices per mouse were counted in the study.

### Cell culture and treatment

We got a mouse microglia cell line (BV-2, BS-C1081185), a human normal astrocyte line (HA1800, BS-C164116) and a mouse dopaminergic neural cell line (SN4741, BS-C163465) from Shanghai Binsui Biotechnology Co., ltd (Shanghai, China). The cells were then cultured in a complete medium containing 89% DMEM, 10% FBS and 1% penicillin–streptomycin. The cells were digested at a specific cell density using 0.05% or 0.25% trypsin. The cells were then passaged or inoculated into culture dishes, 6-well plates, 12-well plates, 24-well plates, or 96-well plates to prepare for later experiments. We transfected BV-2 cells with HCA2-siRNA (SiHCA2, 25 nM) or PPAR γ-siRNA (SiPPAR γ, 25 nM) for 24 h or treated BV-2 cells with 10 μM MK2206 (Akt inhibitor) for 6 h, followed by treatment with NA (1 mM) and stimulated with LPS (100 ng/mL) for 2 h. After replacing the culture medium, the cells would continue to be cultured for 24 h. After collecting the supernatant and mixing it with fresh medium (1:1), we obtained the conditioned medium. SN4741 cells were cultured with the conditioned medium for 24 h.

### Western blotting

Total protein from cells and tissues was extracted using protein lysate (P0013B, Beyotime Inst Biotech, Beijing, China) and aliquoted after concentration determination using the BCA kit according to the manufacturer's protocol (Beyotime Inst Biotech, Beijing, China). After that, we performed western blotting following the previous experimental protocol. In this experiment, the proteins were transferred to polyvinylidene fluoride (PVDF) membranes (Millipore, Billerica, MA, USA)., which were then conjugated with primary antibodies against β-actin (1:5,000, Cat No. 81115–1-RR), IBA-1 (1:800, Cat No. 66827–1-Ig), TH (1:800, Cat No. 66334–1-Ig), AKT (1:2,000, Cat No. 60203–2-Ig), p-AKT (1:500, Cat No. 66444–1-Ig), NF-κB p65 (1:1,500, Cat No. 8242S), p-NF-κB p65 (1:500, 3033S), PPARγ (1:1,500, Cat No. 16643–1-AP), followed by binding to secondary antibodies Goat anti-mouse (1:4,000, BA1050) or Goat anti-rabbit (1:4,000, BA1055). The primary antibody was incubated for 12 h at 4 °C and the secondary antibody was incubated for 2 h at room temperature. All primary antibodies were from Proteintech (Wuhan, China) and Cell Signaling Technology (MA, US). All secondary antibodies were from Boster (Wuhan, China). Finally, according to the manufacturer's protocol, we detected the protein using ECL western blotting Detection Reagents (Millipore, MA, US). The results were analyzed using Image J software.

### ELISA

After dissecting the mice, their midbrain and blood were quickly collected. The midbrain was washed with PBS, after which it was dried and weighed. The tissue was then ground with high-throughput tissue grinder and diluted with PBS (5 μL/mg tissue) containing 0.1% protease inhibitor (Beyotime Inst Biotech, Beijing, China). It was then broken up with an ultrasonic homogenizer and centrifuged (4 °C, 12,000 rpm, 20 min) to obtain the final supernatant. Blood was collected, left to stand and centrifuged (4 °C, 2,500 rpm, 10 min) to get the serum. We measured the levels of IL-6 (Cat^#^ 431,307), IL-1β (Cat^#^ 575,109) and TNF-α (Cat^#^ 430,907) in the serum with reference to the manufacturer's protocol of the ELISA kit (Bio Legend, San Diego, CA, US).

### Transcriptome sequencing

Midbrain tissue samples from mice were obtained. Sequencing and analysis in the experiments were done by Bio Marker Biotechnology Co (Beijing, China). Briefly, RNA from midbrain tissue was extracted, and RNA quality was detected using Nanodrop and Agilent 2100. After the sample is tested as qualified, the sample library is constructed. The process mainly includes: the enrichment of eukaryotic mRNA by magnetic beads with Oligo (dT); fragmentation buffer is then added to interrupt mRNA randomly, the first cDNA strand was synthesized with six base random primers using mRNA as a template, then the second cDNA strand was synthesized by adding buffer, dNTPs, RNase H and DNA polymerase I, and the cDNA was purified with AMPure XP beans. The purified double-stranded cDNA was repaired at the end, the poly-tail was added and connected with the sequencing connector, and AMPure XP beans were used to select the fragment size. Finally, the cDNA library was obtained by PCR enrichment. After the library construction was completed, the quality was tested, and the computer sequencing was carried out only after the test results met the requirements.

### RNA interference

BV2 or SN4741 cells were seeded into 24-well plates at 1 × 10^6^ cells/well density, placed in an incubator, and when the cell density reached about 70%, SiRNA transfection was done according to the instructions. Briefly, siRNA(SiHCA2, 25 nM or SiPPAR γ, 25 nM) and Lipofectamine 2000 (1 μL) (Thermo Fisher Scientific Co., Shanghai, China) were diluted in Opti MEM (100 μL) and left to stand for 5 min at 4 °C, then the two were mixed gently and left to stand for an additional 20 min at 4 °C. After that, the mixture was added to the culture wells and the cells were incubated for 6 h, after which the medium was changed and the culture was continued for 24 h. SiHCA2 and SiPPAR γ were synthesized by Gene Pharma (Shanghai, China).

### Real-time PCR

BV2 cells were starved for 3 h in a serum-free medium, then treated with NA and LPS for 12 h. After that, according to the manufacturer's protocol, we obtained total RNA in BV-2 cells using Trizol reagent (Thermo Fisher Scientific, Shanghai, China). After that, the RNA was reverse transcribed to cDNA with a reverse transcription kit (Roche, Shanghai, China). Next, we detected the mRNA levels using the Quantitect SYBR Green RT-PCR kit (Roche, Shanghai, China). The reaction conditions: the first step 95 °C for 3 min; the second step 95 °C for 10 s; the third step 60 °C for 30 s; the second and third steps were cycled 40 times. Each sample was used in triplicates. Finally, we used the 2^−ΔΔCT^ method to assess the mRNA levels of IL-6, TNF-α, iNOS, COX-2, Ym-1, CD206, Arg-1, and IL-10 relative to β-actin, respectively. Primers were synthesized by Sangon Biotech (Shanghai, China). The sequence refers to previous studies [21, 22]. The primer sequences are shown in Table 1.

**Table 1:** The sequence of the primer

Gene	Forward primer (5ʹ–3ʹ)	Reverse primer (5ʹ–3ʹ)
β-actin	GTCAGGTCATCACTATCGGCAAT	AGAGGTCTTTACGGATGTCAACGT
IL-6	CCAGAAACCGCTATGAAGTTCC	GTTGGGAGTGGTATCCTCTGTGA
TNF-α	CCCCAAAGGGATGAGAAGTTC	CCTCCACTTGGTGGTTTGCT
iNOS	CAACAGGGAGAAAGCGCAAAA	TACTGTGGACGGGTCGATGT
COX-2	TGAGTACCGCAAACGCTTCT	CAGCCATTTCCTTCTCTCCTGT
Ym-1	CAGGGTAATGAGTGGGTTGG	CACGGCACCTCCTAAATTGT
CD206	CTTCGGGCCTTTGGAATAAT	TAGAAGAGCCCTTGGGTTGA
Arg-1	GTGAAGAACCCACGGTCTGT	GCCAGAGATGCTTCCAACTG
IL-10	TGAATTCCCTGGGTGAGAAGC	CATTCATGGCCTTGTAGACACC
HCA2	TGAGGCAGAGACAGATGGACAGAC	GAGAAGCCAGAAGATGCGGATGC
PPARγ	ACAGGAAAGACAACGGACAAATCA	CTTCTACGGATCGAAACTGGCAC

### Immunofluorescence staining

After treatment, BV2 cells were fixed using 4% paraformaldehyde for 10 min at room temperature and permeabilized with 0.1% Triton X-100 for 5 min, then blocked for 4 h with PBS solution containing 5% donkey serum albumin and incubated overnight at 4 °C with primary antibody (iNOS (1:100), Arg-1(1:100) and IBA-1(1:100) (Proteintech, Wuhan, China)) diluted in 5% donkey serum albumin solution. The cells were washed three times with PBS and incubated with the fluorescently labeled secondary antibody (Invitrogen, California, America) in 5% donkey serum albumin solution for 1 h at room temperature. Among them, iNOS and IBA-1 were co-localized, which led to BV2 cells incubated simultaneously with rabbit iNOS primary antibody and mouse IBA-1 primary antibody. After overnight at 4 °C, the cells were incubated with an anti-rabbit fluorescent secondary antibody with a green label and a mouse fluorescent secondary antibody with red label. Arg-1 and IBA-1 were co-localized in the same way. Then the cells were washed three times with PBS and the nuclei were stained with DAPI. Finally, images of the stained sections were obtained using fluorescence microscopy (CX41-32RFL Olympus OLYMPUS).

### Primary microglia extraction

Primary microglia were extracted according to the protocol of a previous study [14]. Briefly, 16–18 days pregnant WT and HCA2^−/−^ mice were euthanized, the whole brains of fetal mice were obtained, and the meninges were removed by visualizing under a stereomicroscope. The cerebral cortex and midbrain were stripped out, placed in 1.5-mL centrifuge tubes and cut into 1-mm^3^ pieces. Subsequently, the tissues were digested for 10 min with 0.25% trypsin. Then the appropriate amount of FBS and DNase1 was added and left for 2 min. After that, it was filtered with a 40-μm filter and centrifuged at 1000 rpm for 3 min. The cell precipitates were added onto poly-l-lysine-coated culture flasks and incubated at 37 °C, 5% CO_2_. After 12 h, the medium was replaced with fresh medium (88% MEM + 10% FBS + 1% penicillin and streptomycin + 1% non-essential amino acids). After that, half-volume fluid changes were performed every 2 days. After 14 days, the culture flasks were placed on a constant temperature shaker at 37 °C for 4 h. Microglia were shaken down and identified by immunofluorescence (Purity of 80% or more).

### Cell counting kit-8 (CCK8) assay

SN4741 cells were digested and put into 96-well plates at 1 × 10^5^ cells/well density. At around 60% cell density, conditioned medium was added. After 24 h, the medium was removed, and CCK8 diluent (Solarbio, Beijing, China) was added to the culture well for further incubation for 3 h. Subsequently, we measured the absorbance at 450 nm with a microplate reader.

### Lactate dehydrogenase (LDH) detection assay

SN4741 cells were digested and put into 96-well plates at 1 × 10^5^ cells/well density. When the cell density reached around 60%, the medium was changed to conditioned medium. After 24 h, the medium was collected and LDH levels were measured according to the instruction using an LDH kit (Beyotime Inst Biotech, Beijing, China).

### Flow cytometry

SN4741 cells were digested and put into 12-well plates at 1 × 10^4^ cells/well density. When the cell density reached around 60%, the medium was changed to conditioned medium. After 24 h, the cells were harvested, and apoptosis levels were measured according to the instructions using an Annexin V-FITC/PI assay kit (Vazyme Biotech, Nanjing, China). Briefly, cells weighing 50,000 to 100,000 were centrifuged at 200 g for 5 min. Then the supernatant was removed, and the cells were gently suspended in 195 μL Annexin V-FITC binding solution. Then 5 μL Annexin V-FITC was added and mixed gently. The cells were centrifuged at 200 g for 5 min at room temperature (20–25 °C) in dark for 10 min, and then the supernatant was removed. After that, 190 μL Annexin V-FITC binding solution and 10 μL propidium iodide staining solution were added to the cells. After the ice bath was kept away from light for 10 min, the apoptosis level was measured by flow cytometry.

### High-performance liquid chromatography (HPLC)

Mice were dissected, and the striatum was acquired. Then HPLC was used to measure dopamine (DA) and its metabolites (DOPAC) in the striatum. Briefly, we placed 0.2 g striatum into a centrifugal tube and added 0.1 M perchloric acid (1 mL), followed by grinding the tissue. After centrifugation (13,000 rpm, 20 min), the supernatant was collected, and sample preprocessing fluid with half the volume (20 mM potassium citrate, 300 mM potassium dihydrogen phosphate and 2 mM edetate disodium) was added to it, followed by centrifugation (13,000 rpm, 20 min). The collected supernatant was filtered with a 0.22 μm filter. Subsequently, the filtrate is put into the high-performance liquid chromatograph to neutralize DA and DOPAC for measurement.

### Data analysis

All experimental data were expressed as mean ± SD. We processed the data using SPSS 19.0 software (IBM) and assessed the differences between groups using a one-way analysis of variance (ANOVA). *p < *0.05* was considered statistically significant. **p < 0.01 and ***p < 0.001 was considered statistically highly significant. Ns was considered no significant difference.

## Result

### HCA2-deficient mice are more prone to PD pathology

We first injected LPS (4 μg/μL, 2 μL) into the midbrain SN of wildtype (WT) and HCA2-deficient (HCA2^−/−^) mice. Two or four weeks after injury, the body weight changes of mice were recorded. Prolonged LPS injury time caused the HCA2^−/−^ mice to undergo more significant body weight loss compared with the WT group (Fig. 1B). Behavioral tests were then performed. The results of the open-field experiment showed that LPS injury for 4 weeks caused the motor and exploration abilities of HCA2^−/−^ mice to decrease significantly compared with WT mice (Fig. 1C–E). Pole climbing experiment showed that the LPS injury for four weeks led to the time required for the HCA2^−/−^ mice to climb the pole being significantly increased compared with WT mice (Fig. 1F). The rotarod test showed that the fatigue resistance of HCA2^−/−^ mice was significantly lower than that of WT mice after LPS injury for four weeks (Fig. 1G). These results collectively indicated that HCA2^−/−^ potentially led to higher chances of dyskinesia in mice after LPS injury.

**Fig. 2:**
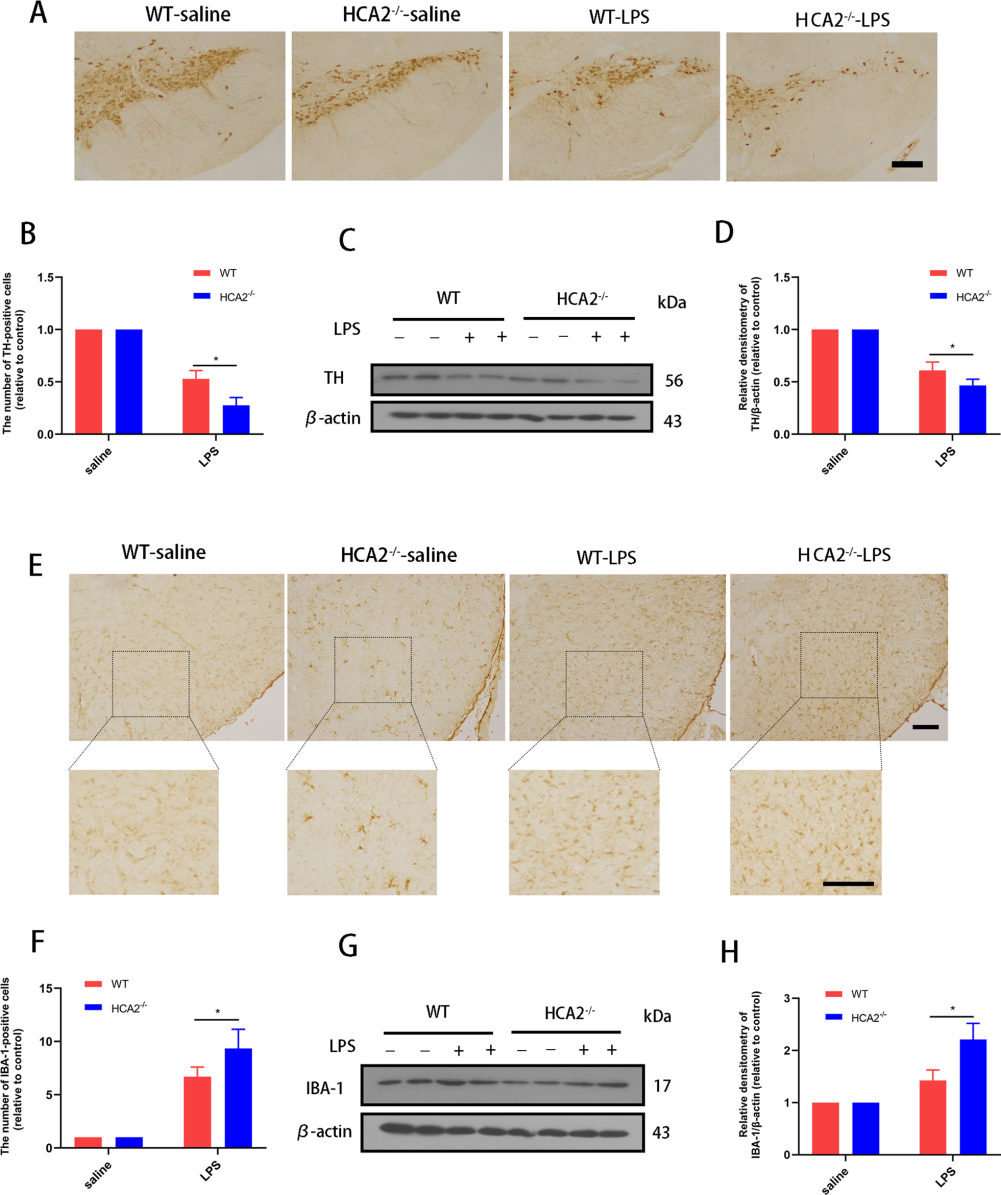
Effect of HCA2 on midbrain dopaminergic neuron injury and microglia activation in mice. **A**, **B** The number of TH-positive cells in the substantia nigra (SN) of the mouse midbrain was detected by immunohistochemistry (the bar represents 50 μm.). **C**, **D** TH protein in mouse midbrain was detected using the western blotting method. **E**, **F** IBA-1-positive cells in the SN of mice were detected using immunohistochemistry (the bar represents 50 μm.). **G**, **H** IBA-1 protein in mouse midbrain was detected using the western blotting method. Results are presented as mean ± SD (*n* = 3). *p < 0.05 means significant difference

The primary pathology of PD is manifest by dopaminergic neuron damage in the midbrain. Therefore, after LPS injury for 4 weeks, we examined the damage to dopaminergic neurons in the midbrain of the two groups of mice. The immunohistochemistry results showed a more severe deterioration of dopaminergic neurons in the SN of HCA2^−/−^ mice compared with the WT group (Fig. 2A, B). Western blotting of tyrosine hydroxylase (TH) proteins in the mouse midbrain also showed consistent results (Fig. 2C, D), indicating that HCA2^−/−^ increases the risk of midbrain dopaminergic neuron damage in mice.

### HCA2-deficient mice are more susceptible to inflammation

LPS is an inflammation-inducing agent that leads to neuronal damage, exacerbating the inflammatory response. Microglia are the primary effector cells of neuroinflammation. The pathological process of PD is accompanied by pro-inflammatory microglia, leading us to test the effect of HCA2^−/−^ on the activation of microglia in the midbrain of mice after 4 weeks of LPS injury. Immunohistochemistry-based analysis revealed more reactive IBA-1-positive cells in the SN of HCA2^−/−^ mice compared with the WT group (Fig. 2E, F). Western blotting of IBA-1 protein in the midbrain also showed the same results (Fig. 2G, H), suggesting that HCA2^−/−^ increased the risk of microglial activation in the mice's midbrain.

**Fig. 3:**
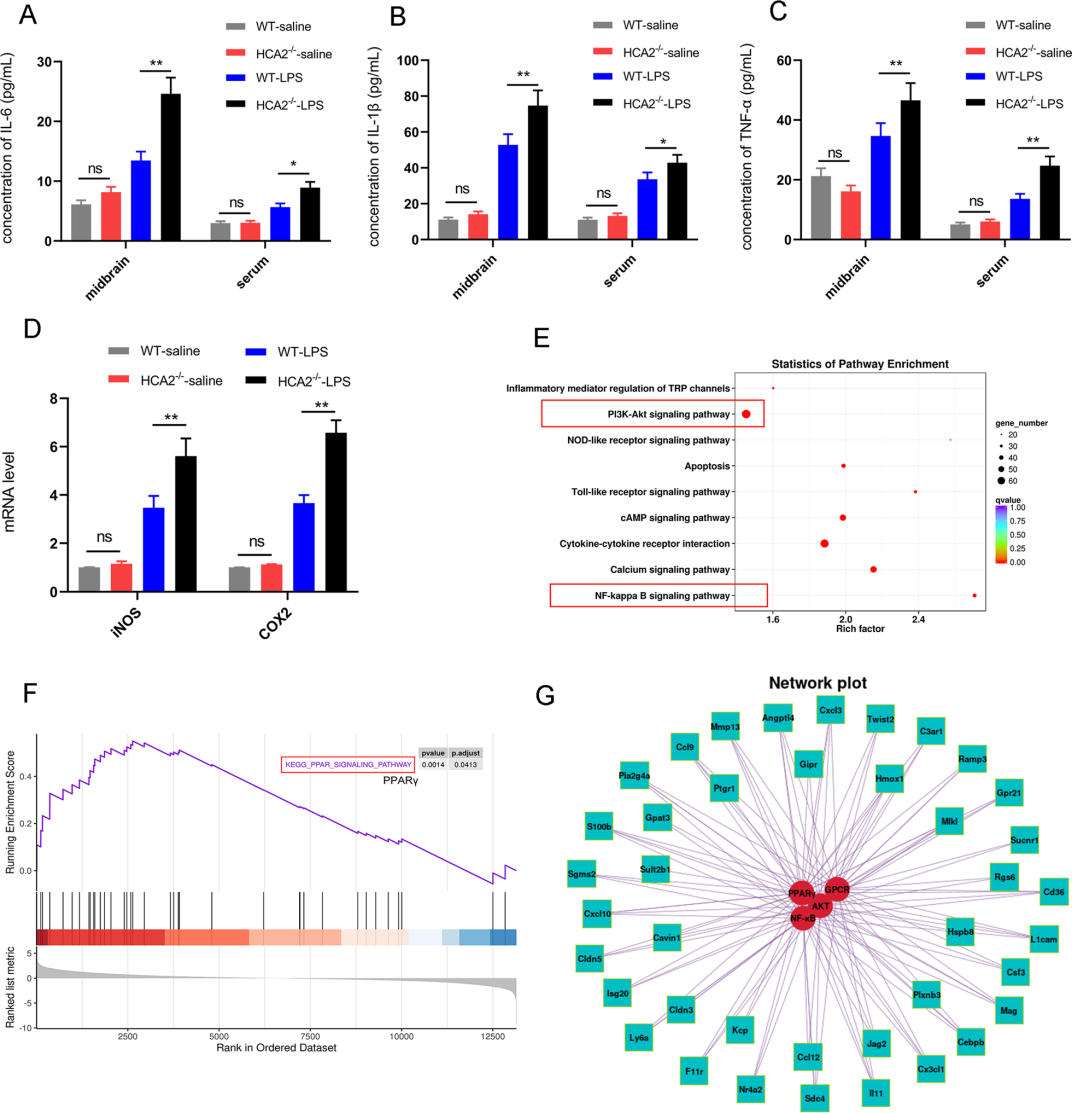
Effect of HCA2 on inflammatory responses in the mice. **A**–**C** The expression of pro-inflammatory mediators in mouse midbrain and serum, as detected using the ELISA method. **D** The mRNA expression of pro-inflammatory protease (iNOS and COX2), as detected using real-time PCR. **E** Pathway enrichment of differential genes between WT mice and HCA2^−/−^ mice in midbrain after LPS injection. **F** GSEA enrichment map of differential genes between WT mice and HCA2^−/−^ mice in midbrain after LPS injection. **G** Network interaction map of differential genes and pathways between WT mice and HCA2^−/−^ mice in midbrain after LPS injection. Results are presented as mean ± SD (*n* = 3). *p < 0.05, **p < 0.01 means a significant difference and ns means not significant

Next, we detected the levels of pro-inflammatory mediators in mouse midbrain tissue and serum by ELISA to evaluate the effect of HCA2^−/−^ on the inflammatory response in mice. This analysis showed that HCA2^−/−^ mice had higher levels of pro-inflammatory factors (IL-6, IL-1β, and TNF-α) in the midbrain and serum after four weeks of LPS injury, compared with WT mice (Fig. 3A–C). At the same time, compared with WT mice, HCA2^−/−^ mice exhibited higher levels of pro-inflammatory protease in the midbrain after LPS injury for 4 weeks (Fig. 3D).

Our previous findings show that HCA2^−/−^ mice are more susceptible to inflammation. We next obtained mouse midbrain tissue and performed whole-genome transcriptome sequencing to elucidate the mechanism of its action, which showed more genes differed between the WT group and HCA2^−/−^ (Additional file 1: Figure S1). Subsequently, we mainly analyzed differences in gene expression upon LPS injection in two types of mice, which showed that compared with WT mice, 1498 genes were up-regulated and 1432 genes were down-regulated in HCA2^−/−^ mice (Additional file 1: Figure S2A, B). We analyzed the differentially expressed genes (DEGs) and identified the disease-related ones. The results showed that in the HCA2^−/−^-LPS group, the expression levels of pro-inflammatory and nerve injury-related genes were higher than those in the WT-LPS group, and the anti-inflammatory and neuroprotective-related genes were lower than those in the WT-LPS group (Additional file 1: Figure S3).

Subsequently, pathway enrichment analysis and GSEA with the DEGs revealed the enrichment of AKT, PPARγ and NF-κB pathways (Fig. 3E, F). Moreover, the DEGs significantly correlated with AKT, NF-κB and PPARγ pathways (Fig. 3G). These results collectively indicated how HCA2^−/−^ affected the expression of inflammation-related genes in the mouse midbrain, which were associated with AKT, PPARγ and NF-κB pathways.

**Fig. 4:**
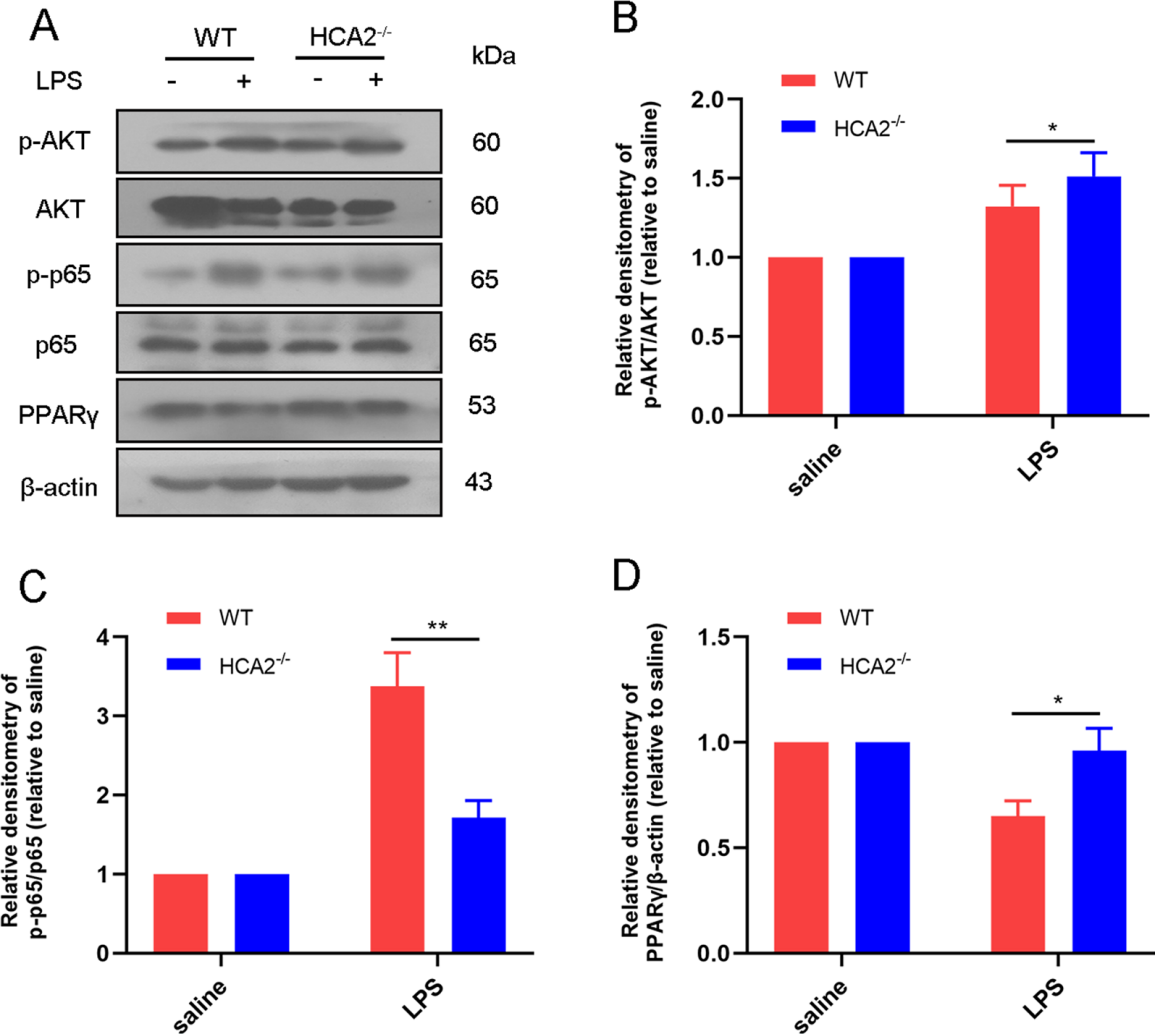
Differences of AKT, PPARγ and NF-κB pathways in the midbrain of WT and HCA2^−/−^ mice after LPS injection. **A**, **B** AKT and its phosphorylated expression. **A**, **C** p65 and its phosphorylated expression. **A**, **D** Expression of PPARγ. Results are presented as mean ± SD (*n* = 3). *p < 0.05, **p < 0.01 means significant difference

We also examined changes in AKT, PPARγ and NF-κB pathways in the midbrain of WT and HCA2^−/−^ mice after LPS injection, which showed the pathways being differentially altered in the brain in WT and HCA2^−/−^ mice, suggesting their involvement in LPS-induced inflammatory responses (Fig. 4).

These results collectively suggest that HCA2^−/−^ increases the susceptibility of mice to inflammation.

### HCA2 regulates the microglial phenotype

We then cultured a microglia cell line (BV2), astrocyte cell line (HA1800), and neuronal cell line (SN4741) in vitro to elucidate the effect of HCA2 on inflammation further. First, we examined the effects of LPS (100 ng/mL) stimulation on HCA2 expression in cells at different time points, which showed that the expression of HCA2 in microglia was significantly higher than in astrocytes and neurons and was more sensitive to LPS (Additional file 1: Figure S4). Then, siRNA-mediated silencing of HCA2 showed significant interference efficiency (Additional file 1: Figure S5). We next treated the three cell lines with an agonist of HCA2 (NA, 1 mM) and evaluated the mRNA levels of pro-inflammatory factors in the cells to investigate the effect of HCA2 on inflammation, which showed the activation of HCA2 primarily affected the inflammatory response of microglia (Additional file 1: Figure S6A–C).

**Fig. 5:**
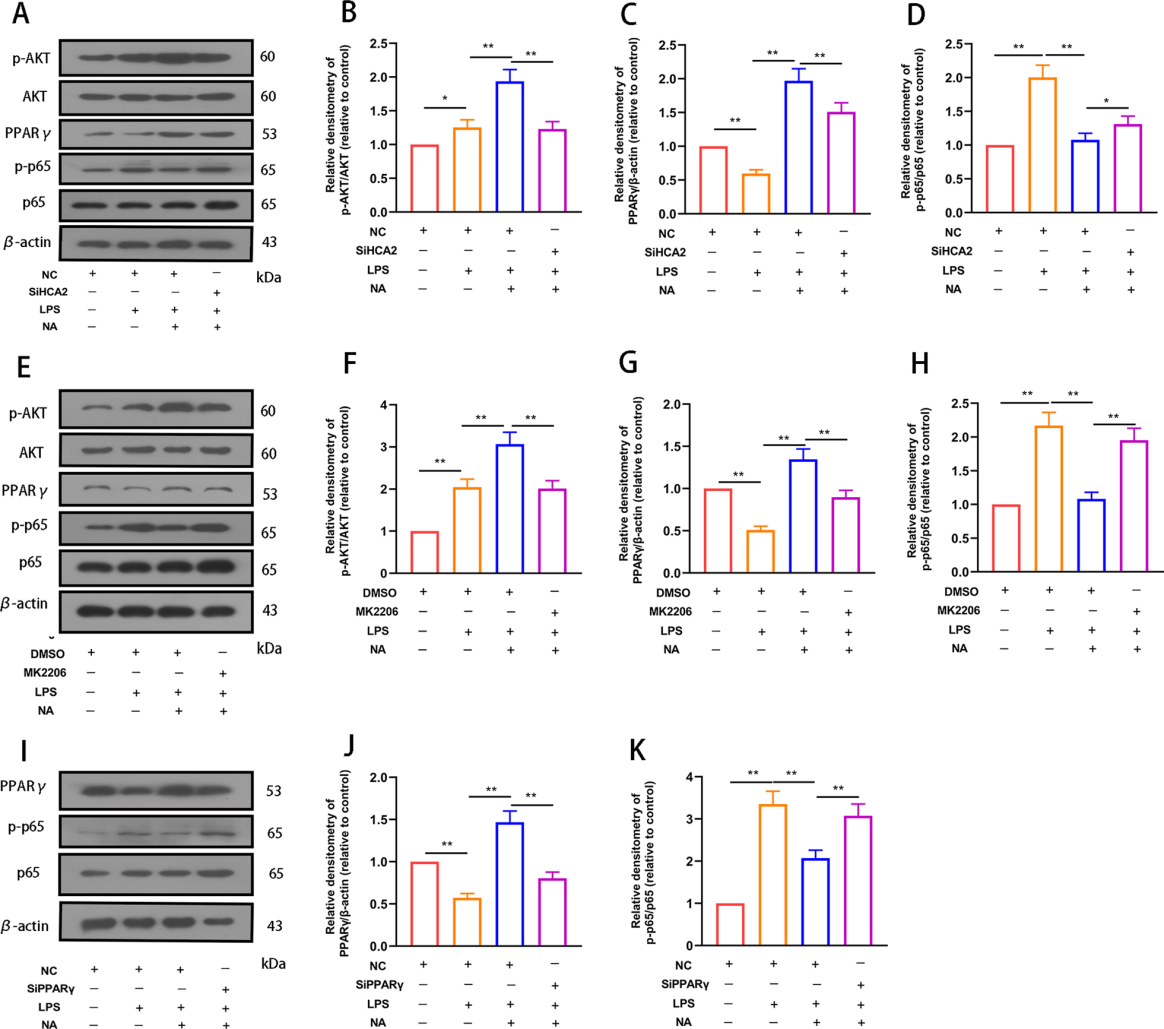
Effect of HCA2 on AKT, PPARγ and NF-κB signaling pathways in microglia. **A**–**D** After BV2 cells were treated with SiHCA2 (25 nM, small interfering RNA of HCA2) for 24 h, the effects of nicotinic acid (NA) on AKT, PPARγ, and NF-κB pathways were examined by western blotting. **E**–**H** After cells were treated with MK2206 (10 μM, AKT inhibitor) for 4 h, the effect of NA on AKT, PPARγ and NF-κB pathways was examined using western blotting. **I**–**K** After cells were treated with SiPPARγ (25 nM, small interfering RNA of PPARγ) for 24 h, the effect of NA on PPARγ and NF-κB pathways was examined using western blot. Results are presented as mean ± SD (*n* = 3). *p < 0.05, **p < 0.01 means significant difference

We next investigated the effect and mechanism of HCA2 activation on microglial inflammation to elucidate further the role played by HCA2. First, we examined the role of HCA2 in regulating AKT, PPARγ, and NF-κB pathways. NA activated AKT and PPARγ pathway and inhibited the NF-κB pathway, and inhibition of HCA2 considerably reversed this effect (Fig. 5A–D), suggesting the need for HCA2 activation. To further clarify the relationship of their mutual influence, we constructed a small interfering RNA of PPARγ (SiPPARγ) and tested its interference efficiency (Additional file 1: Figure S7). MK2206 (AKT inhibitor, 10 μM) and SiPPARγ (25 nM) were added to the cells, which revealed that MK2206 blocked the regulation of NA on the PPARγ and NF-κB pathway (Fig. 5E–H). Also, SiPPARγ blocked the regulatory effect of NA on the NF-κB pathway (Fig. 5I–K), indicating how NA regulates the AKT–PPARγ signaling axis and inhibits the NF-κB pathway by activating HCA2 in microglia.

**Fig. 6:**
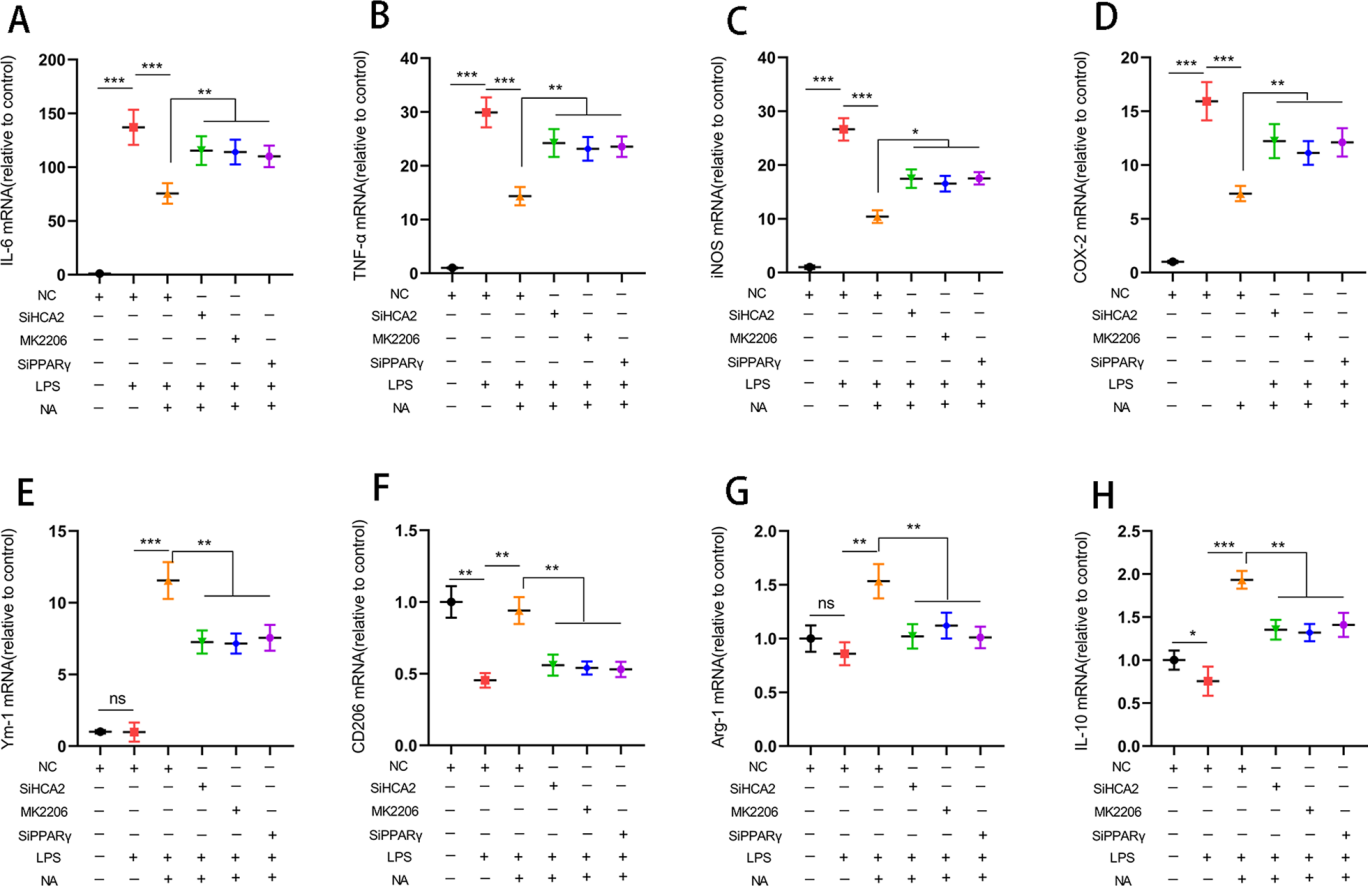
Effect of HCA2 on the expression of pro-inflammatory and anti-inflammatory mediators in the BV2. We, respectively, treated BV2 cells with SiHCA2 (25 nM, 24 h), MK2206 (10 μM, 4 h) and SiPPARγ (25 nM, 24 h), and then treated with NA (1 mM) and LPS (100 ng/mL) for 24 h. After that, we examined the expression of pro-inflammatory mediators [IL-6 (**A**), TNF-α (**B**), iNOS (**C**) and COX2 (**D**) and anti-inflammatory mediators (Ym-1(**E**), CD206 (**F**), Arg-1 (**G**) and IL-10 (**H**)) using real-time PCR. Results are presented as mean ± SD (*n* = 3). *p < 0.05, **p < 0.01, ***p < 0.001 means significant difference and ns means not significant

**Fig. 7:**
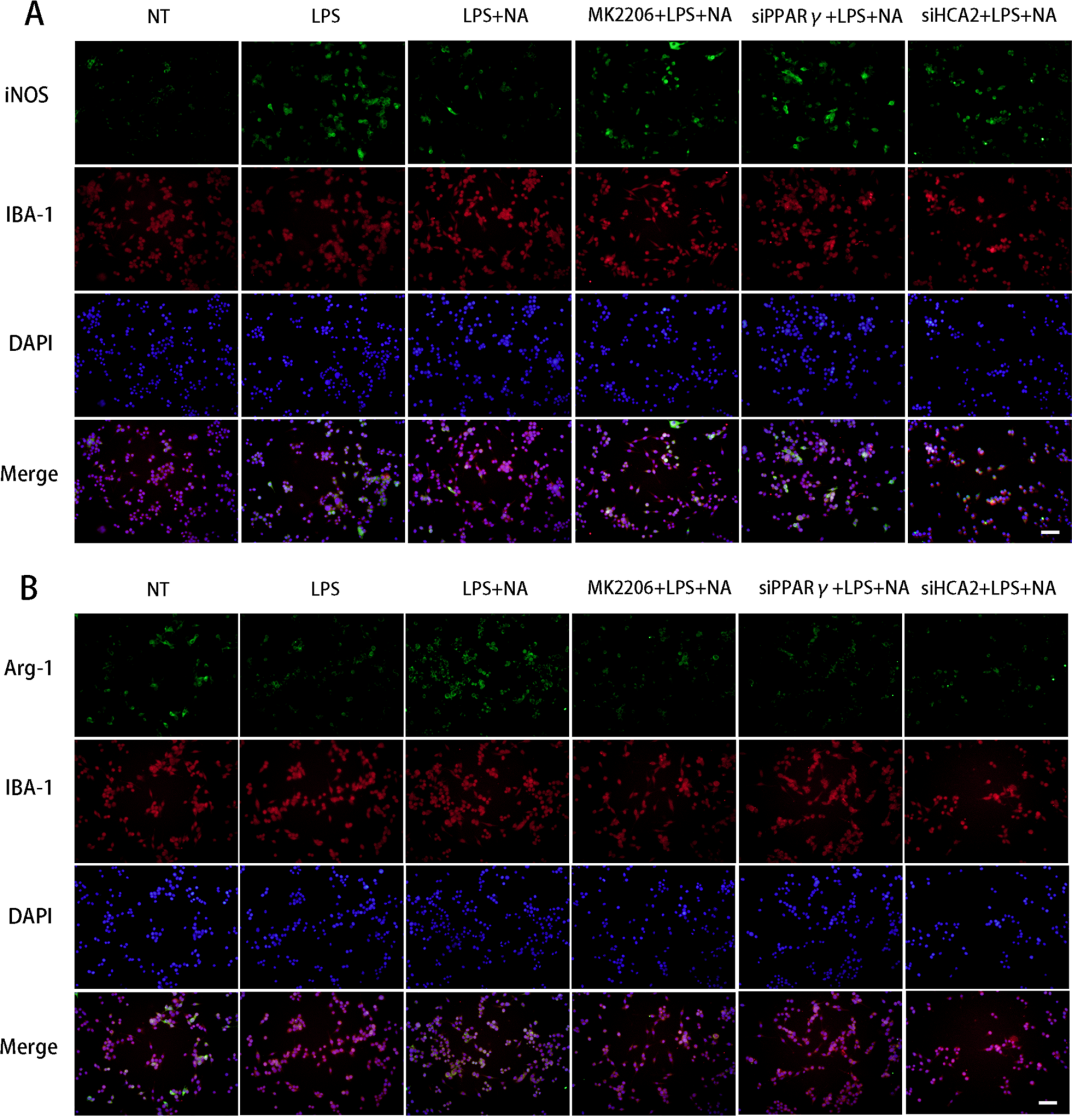
Effect of HCA2 on the expression of iNOS and Arg-1 in microglia. We, respectively, treated BV2 cells with SiHCA2 (25 nM, 24 h), MK2206 (10 μM, 4 h) and SiPPARγ (25 nM, 24 h), and then treated with NA (1 mM) and LPS (100 ng/mL) for 24 h. After that, we examined the expression of iNOS and Arg-1 in microglia. **A** The expression of iNOS was detected using the immunofluorescence staining (the bar represents 20 μm). **B** The expression of Arg-1 was detected using the immunofluorescence staining (the bar represents 20 μm)

Next, we investigated the impact of the above pathways on microglial phenotype, which revealed that NA inhibited the expression of pro-inflammatory mediators [IL-6 (Fig. 6A), TNF-α (Fig. 6B), iNOS (Fig. 6C), COX-2 (Fig. 6D)] in microglia and promoted anti-inflammatory mediators (Ym-1 (Fig. 6E), CD206 (Fig. 6F), Arg-1(Fig. 6G), IL-10 (Fig. 6H)). However, SiHCA2, MK2206 and SiPPARγ treatment modulated this effect (Fig. 6).iNOS and Arg-1 are two key enzymes of the arginine metabolism pathway, where they compete with each other for arginine metabolism substrates during arginine metabolism. They also reflect the polarization phenotype of microglia from the metabolic level. We next evaluated the expression of iNOS and Arg-1 in microglia by immunofluorescence staining to elucidate further the regulatory effect of activated HCA2 on microglia's inflammatory response. NA inhibited the expression of iNOS and promoted the expression of Arg-1, while SiHCA2, MK2206 and SiPPARγ treatment modulated this effect (Fig. 7A, B).

In order to further verify the regulatory effect of HCA2 on microglia inflammation, primary microglia were cultured in vitro and identified by immunofluorescence (Additional file 1: Figure S 8). Next, the changes in mRNA expression of pro- and anti-inflammatory mediators were investigated in WT and HCA2^−/−^ primary microglia after adding NA and LPS further to validate the role of activated HCA2 in anti-neuroinflammation The results showed that NA inhibited the expression of pro-inflammatory mediators (IL-6 (Additional file 1: Figure S9A), TNF-α (Additional file 1: Figure S9 B), iNOS (Additional file 1: Figure S9 C), COX-2 (Additional file 1: Figure S9 D)) and promoted the expression of anti-inflammatory mediators (Ym-1 (Additional file 1: Figure S9 G), CD206 (Additional file 1: Figure S9 F), Arg-1(Additional file 1: Figure S9 E), IL-10 (Additional file 1: Figure S9 H)) in WT primary microglia. At the same time, no significant difference was observed in HCA2^−/−^ primary microglia.

Collectively, these results demonstrate how NA inhibits the expression of pro-inflammatory microglial markers and promotes the expression of anti-inflammatory microglial ones by regulating the HCA2/AKT/PPARγ–NF-κB signaling axis, modulating microglial reactivity.

### NA protects neurons against microglial reactivity-mediated injury by activating HCA2

**Fig. 8:**
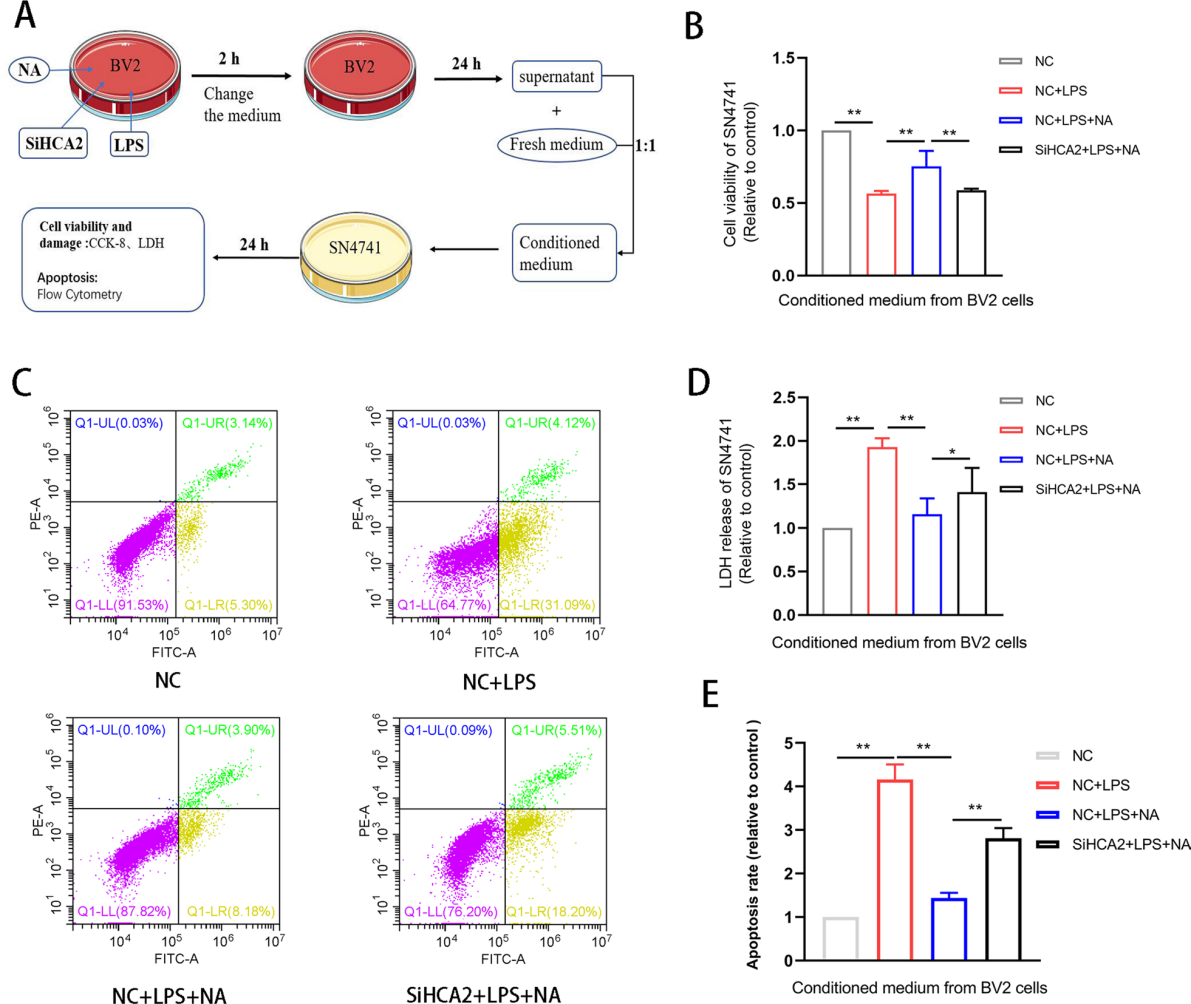
Effect of HCA2 on neuronal injury mediated by microglial activation. **A** Conditioned medium preparation and processing of neurons. **B** Cell viability, as detected using the CCK8 method. **D** LDH release was detected using the LDH method. **C**, **E** Apoptosis of cells was detected using flow cytometry. Results are presented as mean ± SD (*n* = 3). *p < 0.05, **p < 0.01, ***p < 0.001 means significant difference

During PD progression, a persistent inflammatory response mediates nerve damage. Thus, protecting neurons against this damage is essential in PD to further elucidate the neuroprotective role of HCA2. First, SiHCA2-transfected BV2 cells were treated with NA (1 mM) and LPS (100 ng/mL), conditioned medium was collected, and SN4741 cells were cultured with this medium (Fig. 8A). Afterward, analyzing the survival and damage of SN4741 cells showed that NA treatment increased neuronal survival (Fig. 8B) and inhibited microglia-mediated neuronal damage (Fig. 8C–E), whereas SiHCA2 therapy enhanced the effect (Fig. 8B–E). These results suggest that NA protects neurons against microglial reactivity-mediated injury by activating HCA2.

### NA protects against PD symptoms by activating HCA2 in microglia

Previously, we demonstrated that HCA2 affects PD pathology and that NA exerts neuroprotective effects in vitro by activating HCA2 to modulate microglial phenotype. We next constructed a conditional knockout mouse (HCA2^Floxp^cx3cr1^cre^) that knocked out HCA2 in microglia to study the ameliorating effect of NA on PD in vivo. We bred mice, specifically knocking out HCA2 on microglia, using HCA2^Floxp^ mice and Cx3cr1^cre^ mice (Additional file 1: Figure S10). First, we identified floxp and Cre genes and obtained HCA2^Floxp^cx3cr1^cre^ mice expressing floxp and cre genes (Additional file 1: Figure S11). Subsequently, we extracted HCA2^Floxp^cx3cr1^cre^ mice microglia and determined the expression of HCA2 in microglia. The results showed that HCA2 was almost not expressed in the microglia of HCA2^Floxp^cx3cr1^cre^ mice (Additional file 1: Figure S12).

**Fig. 9:**
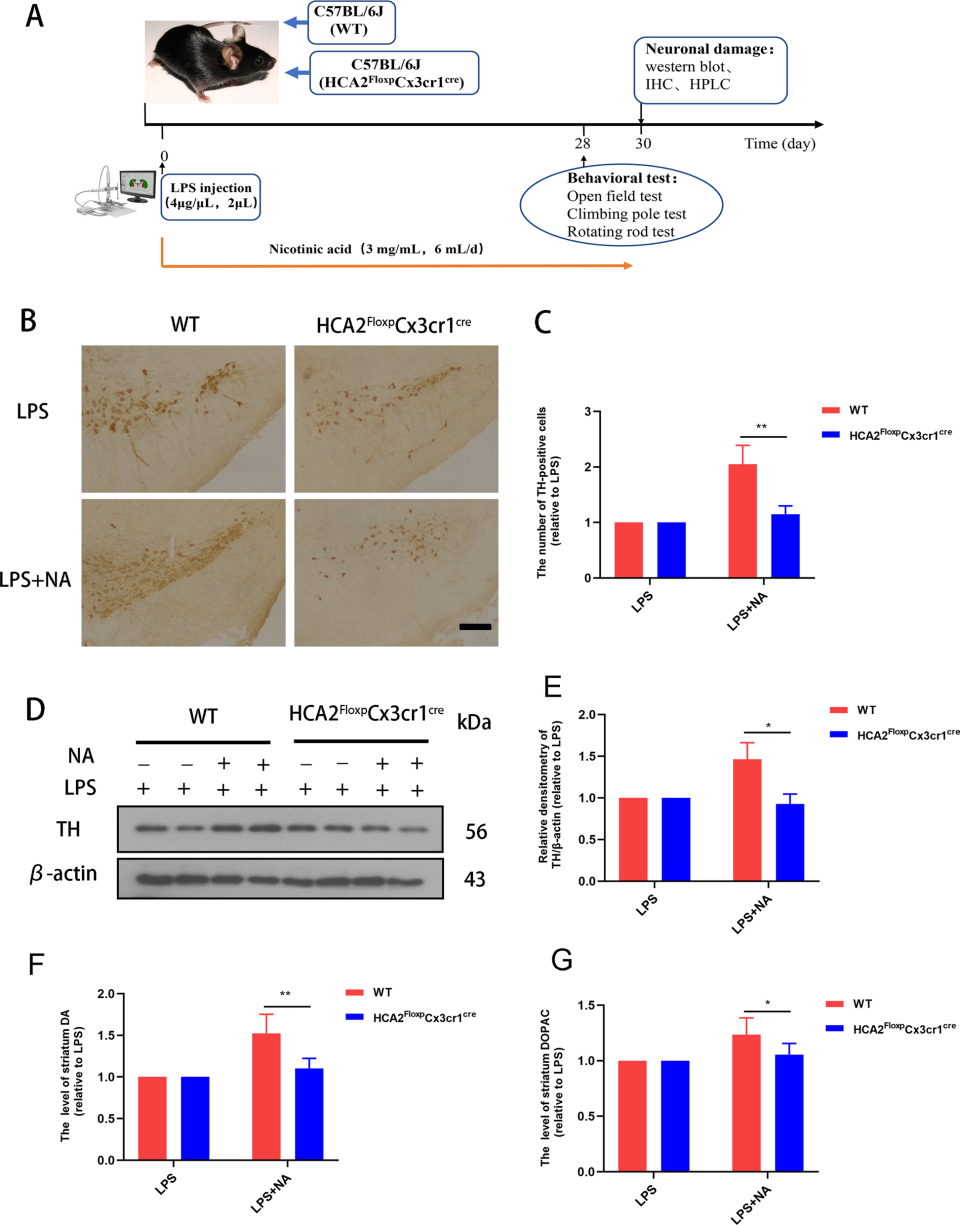
Effect of nicotinic acid (NA) on midbrain dopaminergic neuron damage and striatal dopamine levels in LPS-injected mice in the presence or absence of HCA2 on microglia. **A** Mouse handling flowchart. **B**, **C** The number of TH-positive cells in the substantia nigra (SN) of the mouse midbrain, as detected by immunohistochemistry (the bar represents 50 μm). **D**, **E** TH protein in mouse midbrain, as detected using the western blotting method. **F**, **G** The levels of dopamine and its metabolites (DOPAC) in mouse striatum, as detected by HPLC technique. Results are presented as mean ± SD (*n* = 3). *p < 0.05, **p < 0.01, ***p < 0.00 means significant difference

Subsequently, we studied the ameliorating effect of NA on PD in HCA2^Floxp^cx3cr1^cre^ mice (Fig. 9 A). First, we examined the effect of NA on dopaminergic neurons in the midbrain of mice, which showed that upon feeding with NA, the neuronal damage in the WT mice was significantly ameliorated compared to the HCA2^Floxp^cx3cr1^cre^ group (Fig. 9B–E). At the same time, we detected dopamine (DA) and its metabolites (DOPAC) in the mouse striatum using the HPLC, revealing how the feeding of NA increased DA and DOPAC content in the striatum of WT mice compared with the HCA2^Floxp^cx3cr1^cre^ group (Fig. 9F, G).

**Fig. 10:**
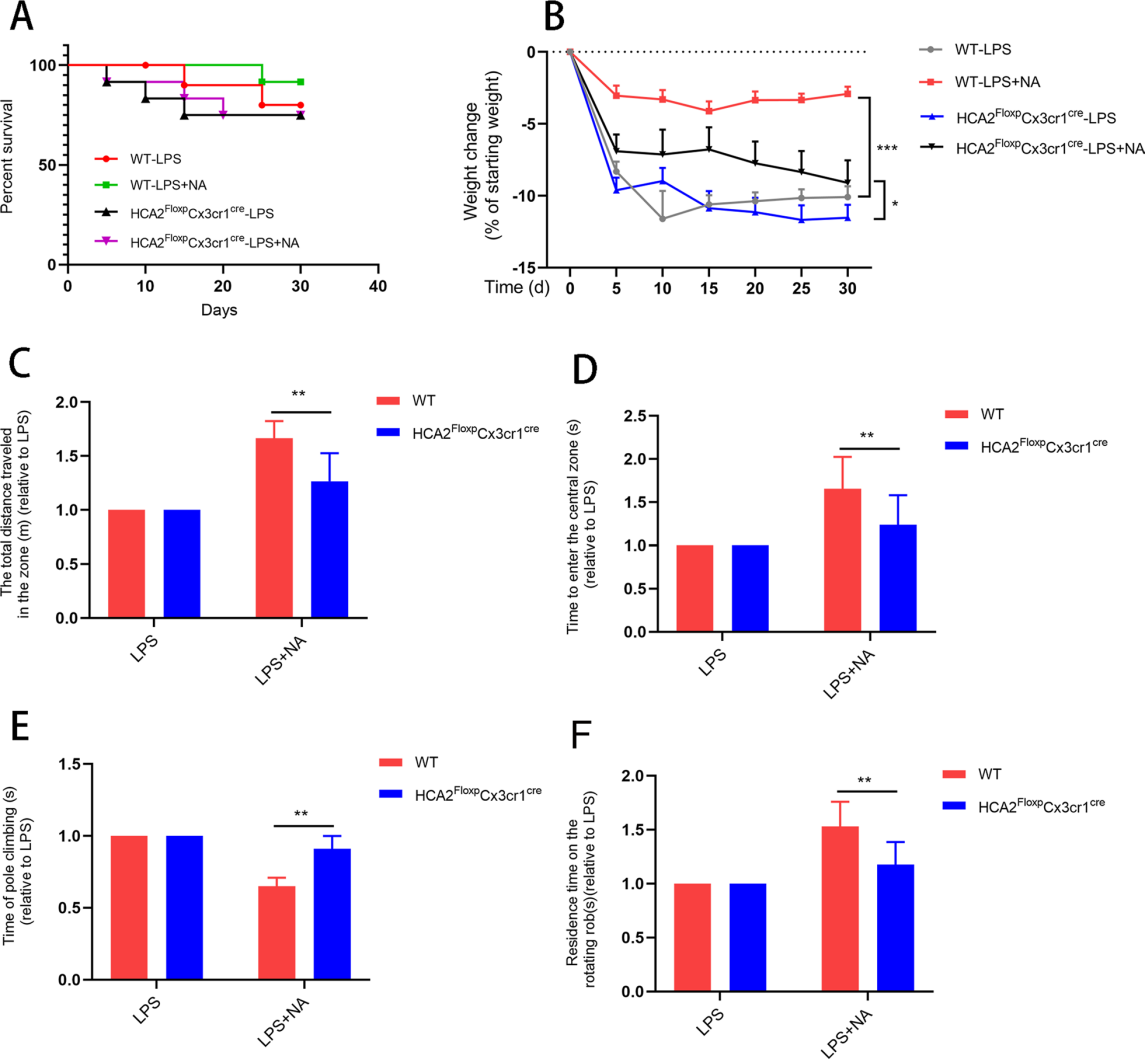
Effect of nicotinic acid (NA) on body weight loss and exercise capacity in LPS-injected mice in the presence or absence of HCA2 in microglia. **A** The survival of mice during the experiment. **B** Changes in body weight of mice during the experiment. **C**, **D** Total distance mice traveled in the open field and time to enter the center box by open-field experiment. **E** Changes in the time it takes for mice to climb the pole through the pole-climbing experiment. **F** Changes in the time mice persisted on the fatigue apparatus through the rotarod experiment. Results are presented as mean ± SD (*n* = 6). *p < 0.05, **p < 0.01, ***p < 0.001 means significant difference

In addition, during the experiment, we also examined mouse survival, body weight loss, and locomotor activity. The results showed that the mortality and body weight loss of WT mice were significantly ameliorated compared with the HCA2^Floxp^cx3cr1^cre^ group upon NA treatment (Fig. 10A, B). Moreover, behavioral tests also demonstrated the beneficial effect of HCA2 in microglia (Fig. 10C–F).

Subsequently, we examined the changes in AKT, PPARγ, and NF-κB pathways in WT mice and HCA2^Floxp^cx3cr1^cre^ mice. The results showed that the activation of AKT and PPARγ and the inhibition of the NF-κB pathway by NA were more pronounced in WT mice compared with HCA2^Floxp^cx3cr1^cre^ mice (Additional file 1: Figure S13). These results validated the regulatory role of HCA2 activation on AKT, PPARγ and NF-κB pathways in vivo.

These results suggest that NA ameliorates PD symptoms in LPS-injected mice by activating HCA2 in microglia.

## Discussion

HCA2 is an orphan GPCR predicted to be responsible for the biological effects of NA. As a cell-surface transmembrane receptor, HCA2 is well known to be abundantly expressed in immune cells. At the same time, its role in immune-related diseases has also been extensively studied. Several previous studies reported that HCA2 plays a role in inflammatory diseases such as enteritis and mastitis [12, 17, 23]. Similarly, the role of HCA2 in neurodegenerative diseases has also been reported [18, 24, 25]. However, its more precise mechanism needs to be further explored. As the second most prevalent neurodegenerative disease that plagues human health, the etiology of PD is still unclear. However, it is well known that patients with PD suffer from massive loss of midbrain dopaminergic neurons and exhibit motor deficits [26, 27]. In this experiment, we found that HCA2 deficiency increased the risk of midbrain dopaminergic neuron damage and decreased exercise capacity in mice. Studies have shown that neuro-immune and inflammatory responses are critical characteristics of PD. PD induces a local inflammatory persistent response in the brain, damaging neurons and aggravating the disease process [8, 28, 29]. In the present study, we found that HCA2 deficiency increased the sensitivity of mice to inflammatory responses, inhibiting which may be one reason underlying HCA2 ameliorating PD pathology. We further performed transcriptome analysis on mouse midbrain tissue, which revealed how the presence or absence of HCA2 affected the expression of disease-related genes, especially the inflammation-related ones in mouse midbrain tissue. We further analyzed DEGs mainly enriched in inflammation-related pathways such as AKT, PPARγ, and NF-κB.

The body's brain homeostasis is based on interactions between all cell types, with microglia and astrocytes serving a wide range of significant neuronal functions [30, 31]. Under normal conditions, microglia and astrocytes also participate in inflammatory responses by acting as local immune cells. During any disruption or loss of homeostasis, microglia are activated, changing their morphology and phenotype, and increasing their motility and phagocytic capacity [32, 33]. Microglia, the primary effector cells of neuroinflammation, is the CNS's first and most important line of immune defense [34, 35]. Our study found that HCA2 was abundantly expressed in microglia and significantly correlated with inflammation. Further research showed that activation of HCA2 effectively inhibited the inflammatory response of microglia.

Studies have shown that microglia have macrophage properties and can be activated to exhibit anti-inflammatory and pro-inflammatory phenotypes. Pro-inflammatory microglia are macrophages that release pro-inflammatory cytokines with strong microbial killing properties, but with specificity to tissue damage. Anti-inflammatory microglia are macrophage cells that produce anti-inflammatory and tissue repair factors, alleviate the inflammatory process and impart organizational protection [36–38]. In this study, our findings suggest that HCA2 activation promoted microglia to achieve anti-inflammatory phenotypes and inhibited pro-inflammatory phenotypes. Our previous research found that AKT and NF-κB pathways were involved in the phenotypic regulation of microglial cells [20]. We explored the mechanism of HCA2 regulating microglial phenotype by investigating the roles of HCA2-affected inflammatory genes and the pathway enrichment patterns. We found that the HCA2 agonist, NA, inhibited the expression of pro-inflammatory microglial markers and promoted the expression of anti-inflammatory ones by regulating the HCA2/AKT/PPARγ–NF-κB signaling axis, thereby modulating microglial inflammation. Notably, LPS stimulation alone in microglia also increased the phosphorylation level of AKT. This suggests multiple roles for the AKT pathway in the inflammatory response and provides a point of interest for future experiments.

It is well known that different polarized types of macrophages and their secreted factors affect the body's energy metabolism. Additional activation of various enzyme pathways leads to arginine metabolism changes in systemic tissue, including the brain. Arginine-1 (Arg-1) is a typical marker of M2 macrophage activation, participating in arginine metabolism. Specifically, the expression of Arg-1 and induced nitric oxide synthase (iNOS) in the CNS use arginine as the only substrate for the biosynthetic pathway. The maintenance of Arg-1 high expression guided arginine metabolism, tending to produce proline or polyamine and NO generation at a low level, which helps neuroprotection [39–41]. Our study revealed that NA regulates the release of Arg-1 and iNOS in microglia by activating HCA2, suggesting that HCA2 might affect PD by affecting arginine metabolism. However, these possibilities remain to be further explored.

During PD, excessively activated microglia release a large number of inflammatory mediators, leading to the damage of dopaminergic neurons, thereby aggravating the pathology of PD [6, 42]. Our previous research proved that HCA2 activates AKT/PPAR γ and inhibits the NF-κB pathway to inhibit microglial inflammation. In vitro models allow for a more in-depth exploration of neuronal protective mechanisms. We used a conditioned medium of microglial cells to treat neuronal cell lines (SN4741) and detected the damage to neurons. Results suggested that activating HCA2 protects neurons against microglial reactivity-mediated injury. These results prompted the role of HCA2-dependent modulation of AKT/PPAR γ/NF-κB signaling in PD pathogenesis.

Given the etiology of its recurrence and the unclear pathogenesis, there is no cure for PD to date. Currently, the clinical treatment of PD primarily involves increasing dopamine release, such as levodopa. However, there are problems such as drug resistance and side effects of long-term use [43–45]. In this study, based on the anti-inflammatory effect of HCA2, we investigated the therapeutic effect of its agonist NA in LPS-injected mice. Our results demonstrate that NA inhibits inflammatory response and ameliorates PD pathology in mice by activating HCA2 on microglia, which suggests that NA has the potential to be a candidate drug for PD treatment.

## Conclusions

**Fig. 11:**
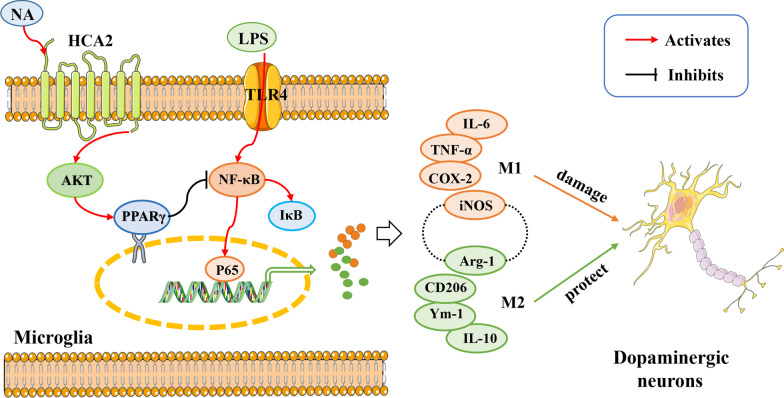
The niacin receptor HCA2 modulates microglial phenotype to inhibit neurodegeneration in LPS-induced vivo and vitro model

In this study, we found that HCA2 deficiency increased the susceptibility of mice to PD pathology and inflammatory responses. In further studies, the results showed that this was closely related to microglia. Subsequently, mechanistic studies confirmed the activation of HCA2 in microglia modulated microglial polarization phenotype through the AKT/PPARγ–NF-κB signaling axis. We further validated that NA significantly alleviated PD pathology in mice by activating HCA2 in microglia. The results from our study collectively demonstrate that the niacin receptor HCA2 modulates microglial phenotype to inhibit neurodegeneration in LPS-induced in vivo and in vitro models (Fig. 11). Our study suggests that neuroinflammation and inflammation-related neuronal damage might be important therapeutic targets for PD, also, HCA2 might be a target for screening PD drug candidates by targeting neuroinflammation.

## Supplementary Information

**Additional file 1. **Supplementary figure.

## Data Availability

All data are available in the manuscript.

## Abbreviations

PD: Parkinson's disease
HCA2: Hydroxy-carboxylic acid receptor 2
NA: Nicotinic acid
AKT: Protein kinase B
NF-κB: Nuclear factor kappa-B
PPARγ: Peroxisome proliferator-activated receptor γ
Arg-1: Arginine-1
iNOS: Induced nitric oxide synthase
CD206: Macrophage mannose receptor
Ym-1: Chitinase 3-like 3
IL-6: Interleukin-6
TNF-α: Tumor necrosis factor-α
COX2: Cyclooxygenase-2
CNS: Central nervous system
SN: Substantia nigra
ANOVA: Analysis of variance
