# Supplementary material for: Activation of HCA2 regulates microglial responses to alleviate neurodegeneration in LPS-induced in vivo and in vitro models
Source: J Neuroinflammation. 2023 Mar 29;20:86. doi: 10.1186/s12974-023-02762-5 (PMC10053461; doi:10.1186/s12974-023-02762-5)
Supplement: Supplementary file 1 — Additional file 1. Supplementary figure. [file 12974_2023_2762_MOESM1_ESM.pdf]

Supplementary figure

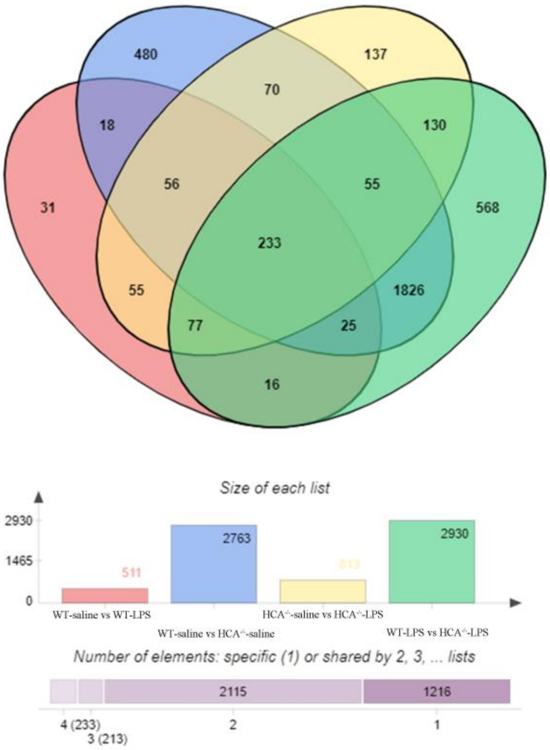

Supplementary figure1. Venn diagram of differential genes between different groups.

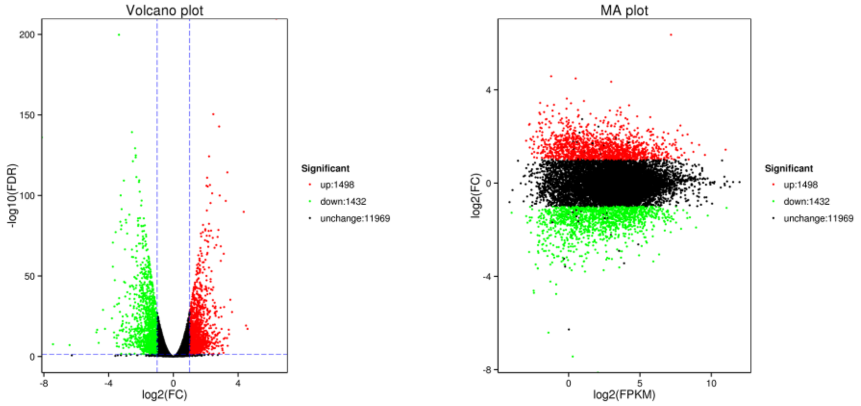

Supplementary figure2. Volcano and MA diagram of differential genes between WT and HCA2$^{-/-}$ groups.

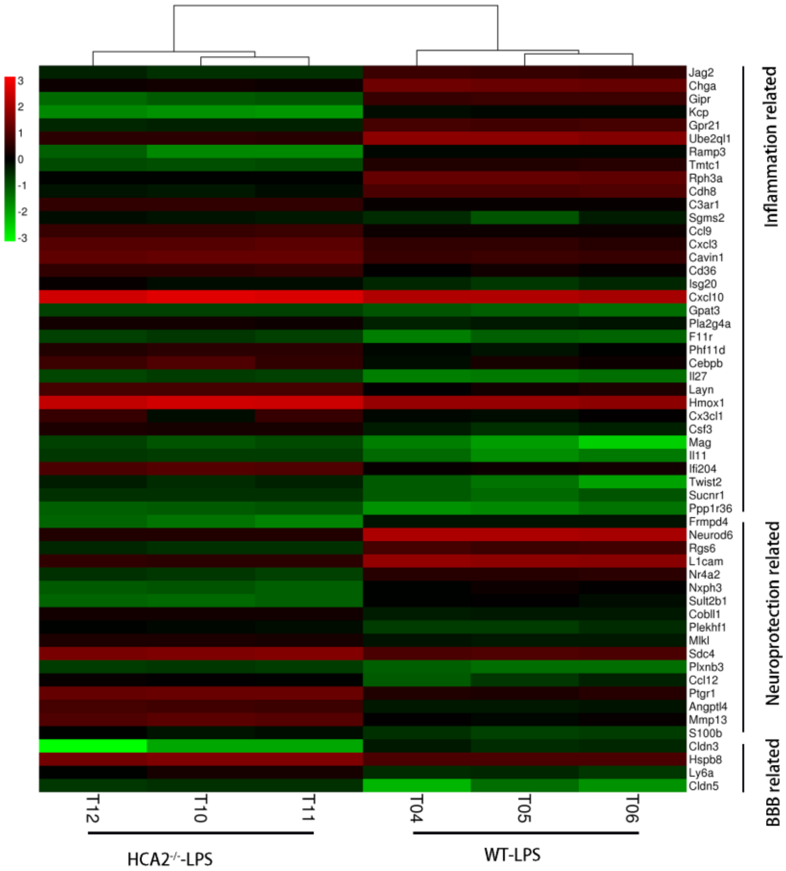

Supplementary figure3. Heatmap of differential genes between WT and HCA2$^{-/-}$ groups.

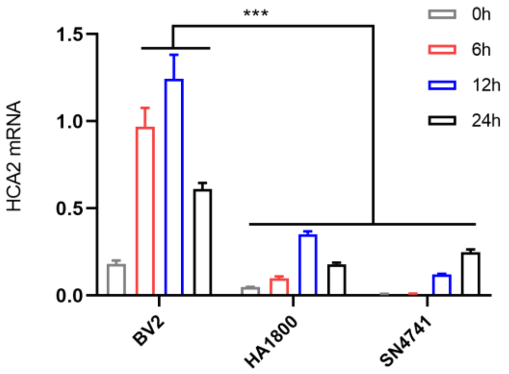

Supplementary figure4. Effects of LPS treatment on HCA2 expression in BV2, HA1800 and SN4741 cells at different times. After LPS stimulation for different times (0 h, 6 h, 12 h and 24 h), the mRNA expression of HCA2 in BV2, HA1800 and SN4741 cells was detected by RT-PCR. ****p<0.001* means significant difference.

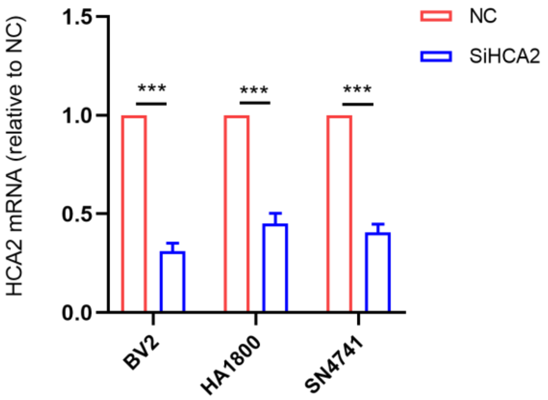

Supplementary figure5. Expression of HCA2 in BV2, HA1800 and SN4741 cells after SiHCA2 transfection. After SiHCA2 transfection for 24 h, the mRNA expression of HCA2 in BV2, HA1800 and SN4741 cells was detected by RT-PCR. ****p<0.001* means significant difference.

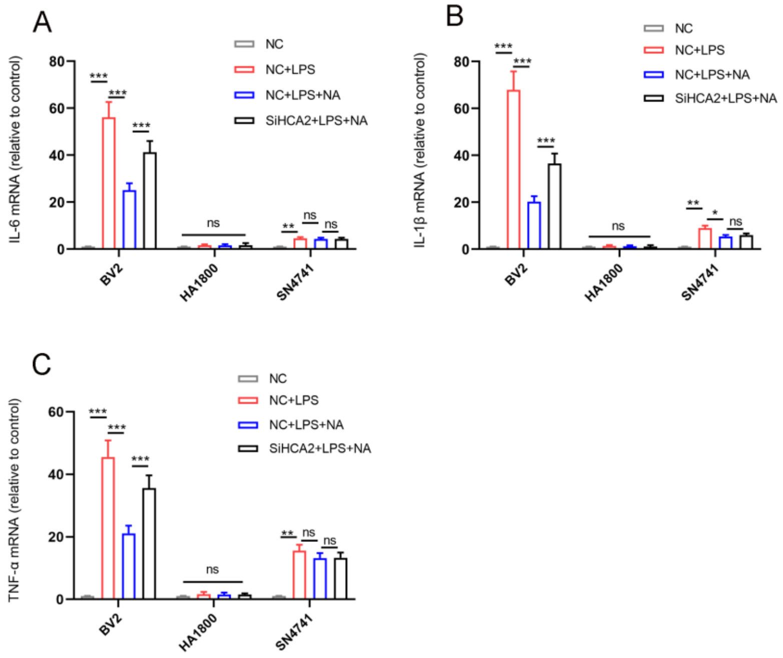

Supplementary figure6. Effects of NA on proinflammatory mediators in BV2, HA1800 and SN4741 cells after SiHCA2 transfection. First, we transfected BV2, HA1800 and SN4741 cells with SiHCA2 (25 nM) for 24 h. Next, we treated three cell lines with HCA2 agonists (NA, 1mM) and LPS (100 ng/mL) for 12 h, and then detected the mRNA level of proinflammatory factors in the cells by real-time PCR. **p<0.05, **p<0.01* and ****p<0.001* means significant difference.

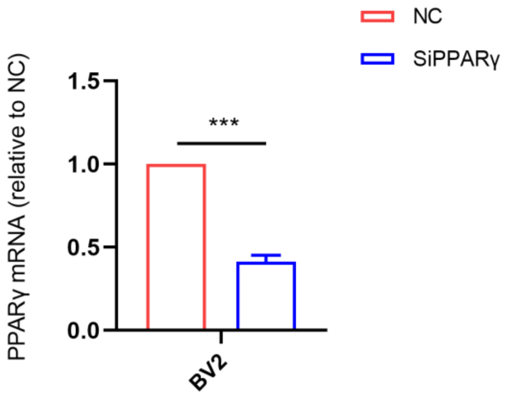

Supplementary figure7. Expression of PPARγ in BV2 cells after SiPPARγ transfection. After SiPPAR γ transfection for 24 h, the mRNA expression of PPARγ in BV2 cells was detected by RT-PCR. ****p<0.001* means significant difference.

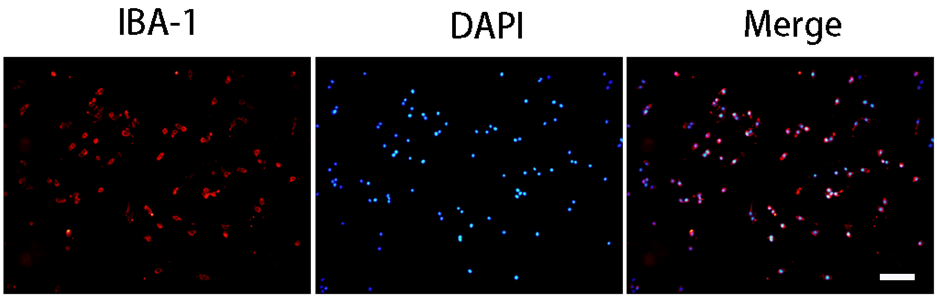

Supplementary figure8. Primary microglia were stained by immunofluorescence staining. The bar represents 50 μm.

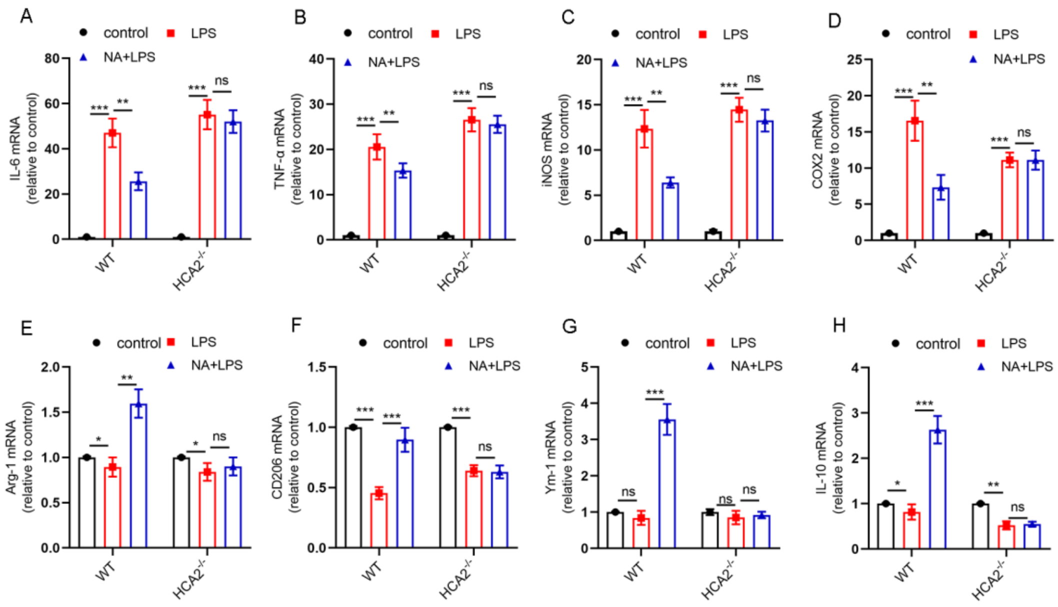

Supplementary figure9. Effect of HCA2 on the expression of pro-inflammatory mediators and anti-

inflammatory mediators in the primary microglia. We respectively treated primary microglia with NA (1mM) and LPS (100 ng/mL) for 24 h. After that, we examined the expression of pro-inflammatory mediators (IL-6 (A), TNF-α (B), iNOS (C) and COX2 (D)) and anti-inflammatory mediators (Arg-1 (E), CD206 (F), Ym-1 (G) and IL-10 (H)) using real-time PCR. Results are presented as mean ± SD (n=3). *$p<0.05$, **$p<0.01$, ***$p<0.001$ means significant difference and ns means not significant.

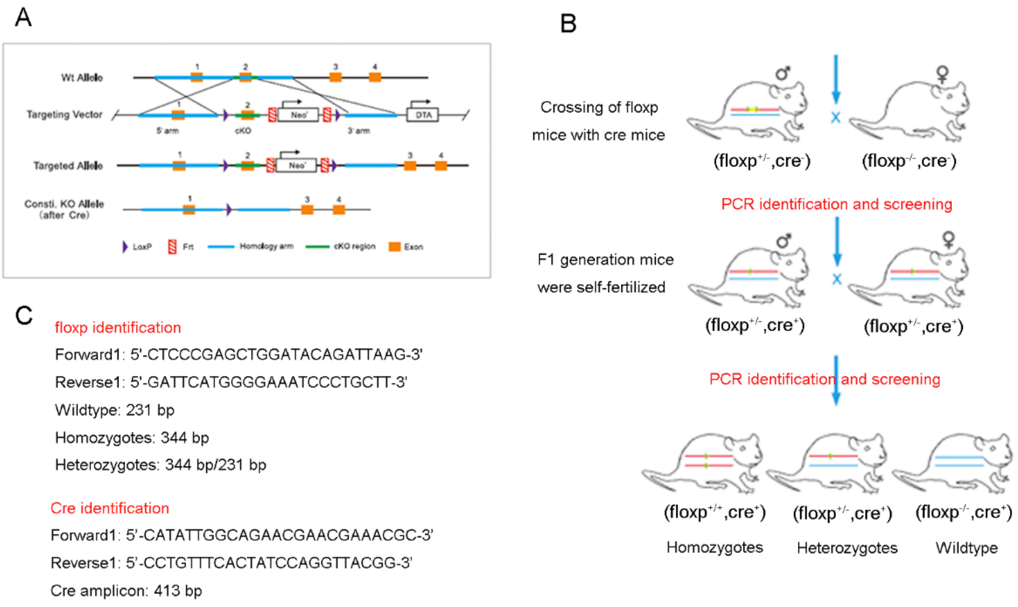

Supplementary figure10. Reproduction and identification protocol of floxp mice and Cre mice. A The insertion position of the floxp locus. B The mating protocol of floxp mice and Cre mice. C Identification protocol of floxp mice and Cre mice.

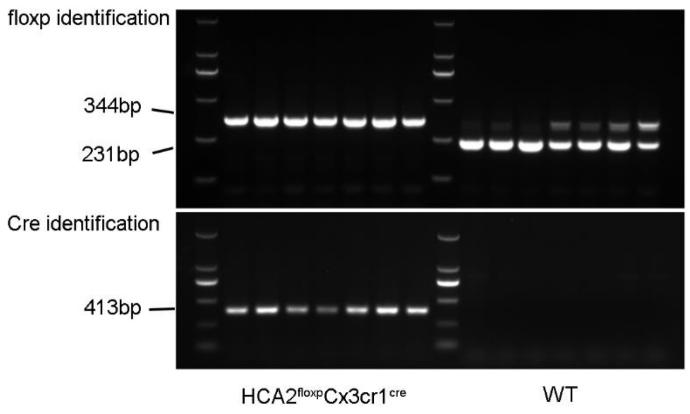

Supplementary figure11. Identification of Floxp locus and Cre gene.

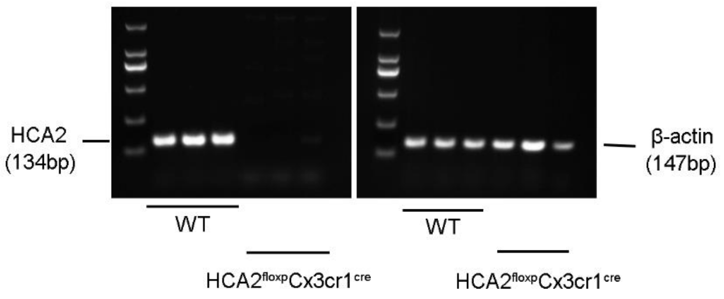

Supplementary figure12. Identification of conditional knockout mice.

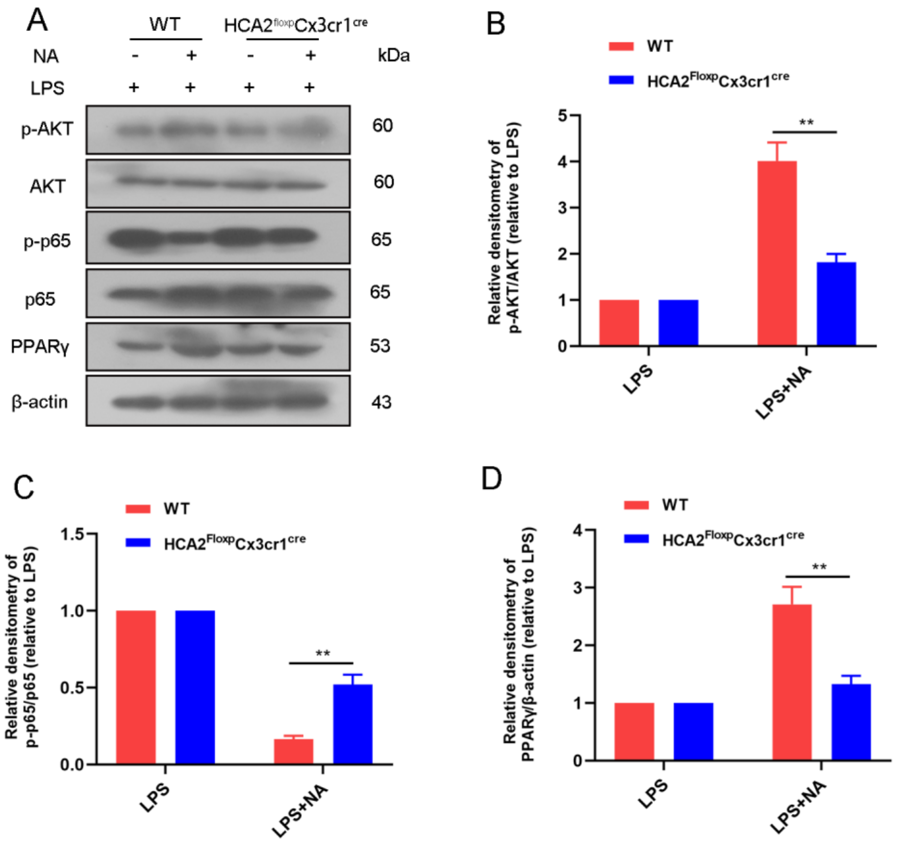

Supplementary figure13. Differences of AKT, PPARγ and NF-κB pathways activations in the midbrain of WT and HCA2^{Floxp}Cx3cr1^{cre} mice after LPS injection and NA administration. A, B: Expression of total and phosphorylated AKT. A, C: Expression of total and phosphorylated p65. A, D: Expression of PPARγ. Results are presented as mean ± SD (n=3). ***p<0.01*means significant difference.
